# Characterization of Split Fluorescent Protein Variants and Quantitative Analyses of Their Self-Assembly Process

**DOI:** 10.1038/s41598-018-23625-7

**Published:** 2018-03-28

**Authors:** Tuğba Köker, Anthony Fernandez, Fabien Pinaud

**Affiliations:** 1 Department of Biological Sciences, University of Southern California, 1050 Child Way, Los Angeles, 90089 California USA; 2 Department of Chemistry, University of Southern California, 1050 Child Way, Los Angeles, 90089 California USA; 3 Department of Physics and Astronomy, University of Southern California, 1050 Child Way, Los Angeles, 90089 California USA

**Keywords:** Biophysical chemistry, Biological fluorescence, Chemical biology

## Abstract

Many biotechniques use complementary split-fluorescent protein (sFPs) fragments to visualize protein-protein interactions, image cells by ensemble or single molecule fluorescence microscopy, or assemble nanomaterials and protein superstructures. Yet, the reassembly mechanisms of sFPs, including fragment binding rates, folding, chromophore maturation and overall photophysics remain poorly characterized. Here, we evolved asymmetric and self-complementing green, yellow and cyan sFPs together with their full-length equivalents (flFPs) and described their biochemical and photophysical properties *in vitro* and in cells. While re-assembled sFPs have spectral properties similar to flFPs, they display slightly reduced quantum yields and fluorescence lifetimes due to a less sturdy β-barrel structure. The complementation of recombinant sFPs expressed *in vitro* follows a conformational selection mechanism whereby the larger sFP fragments exist in a monomer-dimer equilibrium and only monomers are competent for fluorescence complementation. This bimolecular fragment interaction involves a slow and irreversible binding step, followed by chromophore maturation at a rate similar to that of flFPs. When expressed as fusion tags in cells, sFPs behave as monomers directly activated with synthetic complementary fragments. This study resulted in the development of sFP color variants having improved maturation kinetics, brightness, and photophysics for fluorescence microscopy imaging of cellular processes, including single molecule detection.

## Introduction

Split green fluorescent proteins (sGFPs), where symmetric splits of the GFP β-barrel^1^ or strategic removal of one or more of its 11 β-strands^2–6^ are engineered to control the re-assembly of full-length GFPs (flGFPs), provide powerful approaches to study the β-strand structural stability of GFP as well as the photophysics and the photochemistry of its tripeptide chromophore (S65-Y66-G67)^5–9^. Such complementary sGFP fragments can additionally be employed as protein tags to assess the solubility of recombinantly expressed proteins^3^, study protein distributions in cells and animals by ensemble or single molecule fluorescence imaging^10–15^, target nanomaterials in cells^14,16,17^ or design supramolecular protein nanostructures^18,19^. Amongst the various sGFPs available, those based on super-folder GFP^20^ have been particularly useful for the aforementioned applications. This includes the asymmetrically split sGFP 1–10 OPT^3^ (here referred to as sGFPori) and its complementary 11^th^ β-strand peptide (here referred to as M3 peptide), which folds rapidly and forms stable protein fusion tags, undergoes self-complementation without the need for interacting protein partners and can be engineered into yellow (sYFP) or cyan (sCFP) spectral variants for multiplexing^21^. While the complementation of sGFPori or its sYFP/sCFP variants with M3 peptides provide versatile bipartite systems for optical sensing and fluorescence imaging, further improvements of their photophysical properties and better understanding of their folding and self-assembly kinetics are required to generate faster folding protein tags having improved brightness, increased photostability and rapid chromophore maturation. Here we implement a series of site-directed mutations in the amino acid sequence of sGFPori and of its corresponding full-length GFPori (flGFPori) to generate novel flGFP/sGFP variants, flYFPs/sYFPs and flCFPs/sCFPs and study their spectral properties, quantum yield, brightness, fluorescence lifetime, photostability, folding kinetics, chromophore maturation kinetics and fluorescence complementation efficiency, both *in vitro* and in live cells. In particular, we describe a novel variant of sGFPori called sGFP2 that displays improved optical properties for advanced imaging applications such as fluorescence single molecule tracking in live cells by complementation activated light microscopy (CALM)^14,15^.

## Results

### Point mutations to generate full-length and split-GFP, split-YFP and split-CFP variants

The original sGFP 1–10 OPT^3^ (sGFPori) used in this study carries folding reporter GFP substitutions (F64L/S65T/F99S/M153T/ V163A), superfolder GFP substitutions (S30R/Y145F/I171V/A206V) that provides enhanced solubility and increased complementation rate with the 11^th^ β-strand complementary fragment (M3 peptide), and additional substitutions N39I/T105K/E111V/I128T/K166T/I167V/S205T to increase brightness and fluorescence stability upon complementation (Table 1). With the aim of improving further the photophysical properties but also the folding and maturation of sGFPori and full-length GFPori (flGFPori), we introduced a few point mutations around the chromophore and at the protein surface. We used three sequential and cumulative substitutions V167T, S72A and N149K, which have been shown to increase fluorescence brightness, improve folding and provide faster chromophore maturation when associated with L64/T65/T153 in Emerald-GFP^22,23^. Using site-directed mutagenesis we therefore generated, expressed and purified three recombinant variants of flGFPori and sGFPori, namely flGFP1/sGFP1 (V167T), flGFP2/sGFP2 (V167T/S72A) and flGFP3/sGFP3 (V167T/S72A/N149K) (Table 1).

**Table 1:** Fluorescence properties of full-length and split fluorescent protein variants. ε: Molar extinction coefficient, Φ: Quantum yield, ψ: Additional substitutions in flGFPori, δ: Additional substitutions in sGFPori, τ_1_ and τ_2_: Fluorescence lifetimes 1 and 2 for two-photon 870 nm excitation, A_1_ and A_2_: Fractions of fluorescence lifetimes 1 and 2.

	Exc. λ (nm)	Em. λ (nm)	ε (M^−1^ cm^−1^)	Φ	Brightness	τ_1_ (ns)	A_1_ (%)	τ_2_ (ns)	A_2_ (%)	Substitutions
flGFPori	485	507	37,700	0.66	24,880	2.46	100	—	—	S30R, N39I, F64L, S65T, F99S, T105K, E111V, I128T, Y145F, M153T, V163A, K166T, I167V, I171V, S205T, A206V, K221H, F223Y, T225N
flGFP1	485	507	33,800	0.75	25,350	2.5	100	—	—	^ψ^V167T
flGFP2	491	510	38,200	0.77	29,410	2.77	100	—	—	^ψ^V167T, S72A
flGFP3	482	508	39,900	0.68	27,130	2.67	100	—	—	^ψ^V167T, S72A, N149K
flYFP1	511	522	68,300	0.61	41,660	2.76	100	—	—	^ψ^T65G, T203Y, T205S
flYFP2	509	522	38,200	0.54	20,630	1.13	29	3.14	71	^ψ^T65L, T203Y, T205S
flYFP3	515	526	37,100	0.51	18,920	0.72	37	3.59	63	^ψ^T203Y, T205A
flCFP1	435	476	20,400	0.41	8,360	0.98	62	2.57	38	^ψ^D19E, D21E, Y66W, E124V, H148D, T205S
flCFP2	436	476	21,300	0.42	8,950	0.97	27	2.76	73	^ψ^D19E, D21E, Y66W, E124V, H148D, V167I, T205S
sGFPori	485	508	37,700	0.59	22,240	2.29	100	—	—	S30R, N39I, F64L, S65T, F99S, T105K, E111V, I128T, Y145F, M153T, V163A, K166T, I167V, I171V, S205T, A206V
sGFP1	485	508	33,800	0.62	20,960	2.34	100	—	—	^δ^V167T
sGFP2	491	510	38,200	0.67	25,730	2.64	100	—	—	^δ^V167T, S72A
sGFP3	480	508	39,900	0.29	11,600	2.5	100	—	—	^δ^V167T, S72A, N149K
sYFP1	510	523	68,300	0.44	30,100	2.62	100	—	—	^δ^T65G, T203Y, T205S
sYFP2	509	522	38,200	0.09	3,440	0.74	22	2.9	78	^δ^T65L, T203Y, T205S
sYFP3	515	524	37,100	0.35	12,990	0.47	40	3.28	60	^δ^T203Y, T205A
sCFP1	433	475	20,400	0.18	3,670	0.81	40	2.4	60	^δ^D19E, D21E, Y66W, E124V, H148D, T205S
sCFP2	433	476	21,300	0.23	4,900	0.64	25	2.52	75	^δ^D19E, D21E, Y66W, E124V, H148D, V167I, T205S

Based on previous descriptions^20,21^, we also generated a variety of sYFP and sCFP variants. The T203 residue located in close proximity to the GFP chromophore^24^ was replaced by a tyrosine to increase polarizability around the chromophore and induce a red shift of the excitation and emission wavelengths in three flYFP/sYFP variants, flYFP1/flYFP1 (T65G/T203Y/T205S), flYFP2/sYFP2 (T65L/T203Y/T205S) and flYFP3/sYFP3 (T203Y/T205A) (Table 1), whose additional substitutions at residues 65 and 205 provide a good balance between bright yellow fluorescence and spectral separation with GFP^21^. We also produced two flCFP/sCFP variants^21^, flCFP1/sCFP1 (D19E/D21E/Y66W/E124V/H148D/T205S) and flCFP2/sCFP2 (D19E/D21E/Y66W/E124V/H148D/ V167I/T205S) (Table 1). Both variants contain the Y66W substitution that alters the GFP spectral properties to CFP^25^, the H148D and T205S substitution that improve CFP quantum yield (QY)^26,27^, a E124V substitution, which allows for faster folding rates in superfolder GFP^28^ as well as D19E and D21E substitutions that provide faster initial rate of cyan fluorescence appearance compared to sCFP carrying only Y66W and T205S mutations^21^. flCFP2/sCFP2 variants also contain a V167I substitution, which provides increased cyan fluorescence brightness^21^.

### Spectral properties of full-length FP and split-FP variants

**Figure 1:**
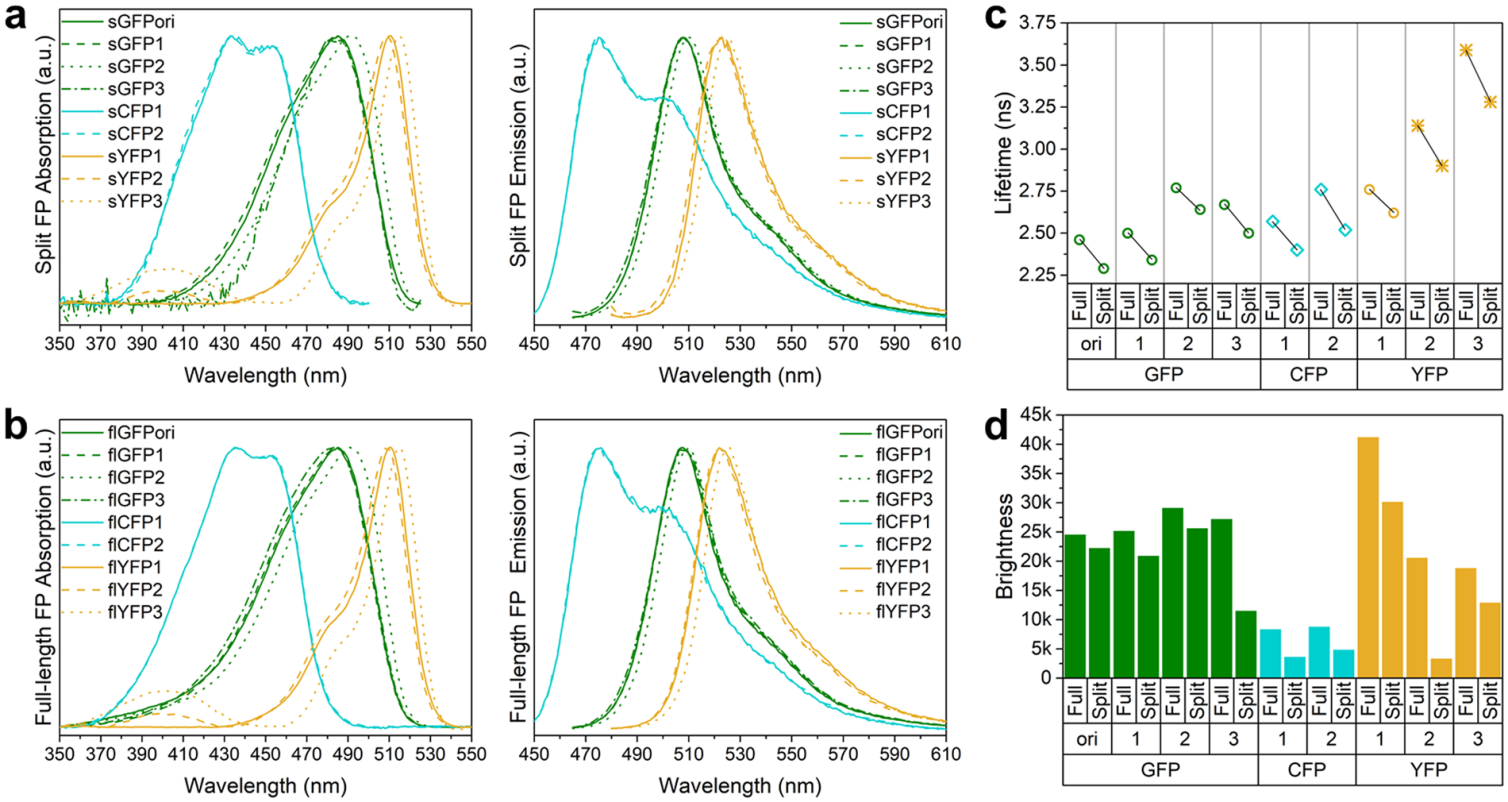
Spectral properties, fluorescence lifetime and brightness of split and full-length fluorescent proteins. (**a**) Absorption and emission spectra of split fluorescent proteins (sFPs). (**b**) Absorption and emission spectra of full-length fluorescent proteins (flFPs). (**c**) Comparison of fluorescence lifetime (τ) between split and full-length fluorescent proteins. ○: lifetime from the chromophore B-state, **◊**: lifetime from the chromophore A-state, *: lifetime from the chromophore I-state. (**d**) Comparison of brightness between all fluorescent proteins.

Complemented sFPs and their corresponding flFPs display similar UV/Vis absorption and emission spectra (Fig. 1a,b). flGFPori/sGFPori and flGFP1/sGFP1 have the same absorption and emission spectra as Emerald-GFP^29^, with absorption maxima at 485 nm and emission maxima at 507 nm. For flGFP2/sGFP2, the additional S72A substitution induces a slight red-shift in both absorption (494 nm) and emission (510 nm) maxima compared to GFPori or GFP1 (Table 1, Fig. 1a,b). However, this S72A-induced red-shift is reversed by the additional N149K substitution in flGFP3/sGFP3. As expected, the T203Y substitution in all the flYFP/sYFP variants effectively results in large spectral red-shifts of the chromophores, which display absorption band maxima around 510 nm and emission maxima around 525 nm (Table 1, Fig. 1a). Interestingly, an additional weaker absorption at 395 nm is observed for flYFP2/sYFP2 and flYFP3/sYFP3, although it is absent in flYFP1/sYFP1 (Fig. 1a,b). This indicates that T65L/T203Y/T205S substitutions in flYFP2/sYFP2, and T203Y/T205A substitutions in flYFP3/sYFP3 modify the chromophore hydrogen-bonding network compared to flYFP1/sYFP1, in accordance with the significant influence that residues 65/203/205 can have on the protonation state of the chromophore in wild-type GFP and its relative absorptions at 395 nm or 470–490 nm^30^. For the flCFP/sCFP variants, the Y66W substitution effectively results in a blue-shift of the absorption and emission spectra, turning GFP into a CFP spectral variant with absorption maxima at 435 nm and emission maxima at 476 nm, as expected. The additional V167I substitution in flCFP2/sCFP2 did not induce significant spectral changes (Table 1, Fig. 1a,b).

### Fluorescence lifetime of full-length FP and split-FP variants

Fluorescence lifetimes (τ) for all the sFP and flFP variants were determined by two-photon frequency domain lifetime measurements at 870 nm excitation. flGFPori/sGFPori and all the flGFP/sGFP variants display single fluorescence lifetime decays around 2.5–2.8 ns (Table 1), consistent with a stabilization of the chromophore benzoidal form (B-state) by amino acids T65 and T203^31^ and the absence of emission from the chromophore quinoidal form (I-state), which normally displays a prolonged fluorescence lifetime at around 3.3 ns^31,32^. The fluorescence lifetime of flGFPori is 2.46 ns, similar to that of Emerald-GFP^33^ but ~0.25 ns shorter than that of S65T-GFP^34^. The V167T substitution in flGFP1 does not significantly affect the fluorescence decay (τ = 2.50 ns). In flGFP2, the S72A substitution results in a slightly longer lifetime of 2.77 ns, which is partially reversed by the additional N149K substitution in flGFP3 (τ = 2.67 ns). Compared to flGFPs, all the complemented sGFPs exhibit ~200 ps shorter lifetime (Fig. 1c, Table 1). This faster fluorescence lifetime decay is likely due to the chromophore being more exposed to the surrounding environment due a less robust protein structure in re-assembled sFPs compared to flFPs, where the chromophore is well protected by the β-barrel. Indeed, complemented sFPs display a higher sensitivity to guanidine hydrochloride denaturation compared to flFPs in equilibrium unfolding assays (Supplementary Fig. S1).

For the YFP variants, the T203Y substitution is expected to induce significant emission from the chromophore I-state with longer fluorescence lifetime decays than for a chromophore B-state emission^32^. Surprisingly, flYFP1 displays a single fluorescence lifetime decay (τ = 2.76 ns), similar to the lifetime expected for a B-state chromophore emission and in agreement with the previously reported lifetime of a S65G/T203V YFP mutant^32^. In comparison, flYFP2 and flYFP3 both have bi-exponential lifetime decays with values around 3.3 ns and 0.5 ns, respectively corresponding to the chromophore I-state and A-state emission, as previously assigned for a T203V/S205A YFP^35^. The additional A-state short fluorescence lifetime^36,37^ in flYFP2 and flYFP3 is consistent with the presence of a residual absorption at 395 nm in these variants. The different fluorescence lifetime behavior between YFP1 and YFP2 underlines the importance of residue G65 in stabilizing the chromophore B-state over A- and I-states when an additional T203Y mutation is present. We note that such stabilization of the chromophore and its possible increased planarity^38,39^ are in agreement with the larger quantum yield and the larger extinction coefficient of YFP1 compared to YFP2 and YFP3 (Table 1). As observed for sGFPs, the complemented sYFPs display slightly shorter fluorescence lifetime than flYFPs and reduced β-barrel stability against denaturant compared to flYFPs (Fig. 1c, Table 1 and Supplementary Fig. S1).

Bi-exponential lifetime decays are also observed for both flCFP variants, in agreement with the two fluorescence lifetimes previously reported for ECFP^40^. The longest lifetimes of flCFP1 and flCFP2 are 2.57 ns and 2.76 ns respectively (Table 1), consistent with the 2.52 ns lifetime of the chromophore A-state induced by the Y66W substitution in ECFP^36,40–43^. The shortest lifetimes of flCFP1 and flCFP2 are 0.98 ns and 0.97 ns respectively (Table 1), in good agreement with the second ~0.6 ns lifetime of ECFP^40^, which is associated with the chromophore B-state conformation. Contrary to previous observations in an ECFP/H148D variant^27^, the H148D substitution present in both flCFP1 and flCFP2 did not result in single exponential fluorescence lifetime decays, indicating that additional substitutions in flCFPs suppress this H148D contribution. Again, complemented sCFPs have shorter fluorescence lifetimes and are more sensitive to chemical denaturation than flCFPs (Fig. 1c, Table 1 and Supplementary Fig. S1).

### Quantum yield and extinction coefficient of full-length FP and split-FP variants

flGFPori has a quantum yield (Φ) of 0.66, slightly higher than EGFP^22^ (Φ = 0.60) and an extinction coefficient (ε) of 37700 M^−1^cm^−1^, similar to that of S65T-GFP^44^ but lower than that of EGFP^45,46^ (ε = 56000 M^−1^cm^−1^), making it overall 25% less bright than EGFP (Table 1). The V167T substitution in flGFP1 increases the quantum yield but additionally reduces the extinction coefficient, such that flGFP1 is only 2% brighter than flGFPori (Fig. 1d and Table 1). However, the additional S72A substitution in flGFP2 further improves both the quantum yield (Φ = 0.77) and the extinction coefficient (ε = 38200 M^−1^cm^−1^), leading to flGFP2 being 20% brighter than the flGFPori and nearly as bright as EGFP (Fig. 1d and Table 1). flGFP2 is the brightest FP amongst the green variants because the additional N149K substitution in flGFP3 results in a lower quantum yield (Φ = 0.68) and an overall decreased brightness (Fig. 1d and Table 1). Quantum yields for the complemented sGFP variants display a similar trend but are generally 10% lower than those of flGFPs, except for sGFP3, which suffered a large loss of quantum yield (Φ = 0.29) (Table 1). Such reduced quantum yields are consistent with the decreased fluorescence lifetimes observed for complemented sGFP variants compared to flGFPs and suggest that the β-barrel surrounding the chromophore in re-assembled sFPs is less rigid than in flFPs, allowing the chromophore to be relatively mobile^47,48^.

Amongst the flYFP variants, flYFP1 has the highest quantum yield with a value of 0.61 similar to that reported for EYFP^22,49^, and the largest extinction coefficient (Fig. 1d and Table 1). This makes it the brightest of all flYFPs, although it remains 20% less bright than EYFP because of a comparatively lower extinction coefficient. The complemented sYFP variants display quantum yields reduced by 30% compared to flYFPs, except for sYFP2 which has a dramatically reduced quantum yield (Φ = 0.09).

The flCFP variants have the lowest extinction coefficients and quantum yields compared to flGFPs and flYFPs. Both flCFP1 and flCFP2 have respective quantum yields of 0.41 and 0.42, values that are similar to that of S65T-ECFP (W1B, Φ = 0.4)^22^. Their extinction coefficients (flCFP1: 20400 M^−1^cm^−1^, flCFP2: 21300 M^−1^cm^−1^, Table 1) however, are significantly lower than for S65T-ECFP (32500 M^−1^cm^−1^)^22^ making them 35% less bright. The complemented sCFP variants display reduced quantum yield compared to flCFPs with losses in the range of 50–60%, indicative of a β-barrel backbone being significant less rigid in re-assembled sCFPs than in flCFPs.

### Photobleaching characteristics of split-GFP variants

**Figure 2:**
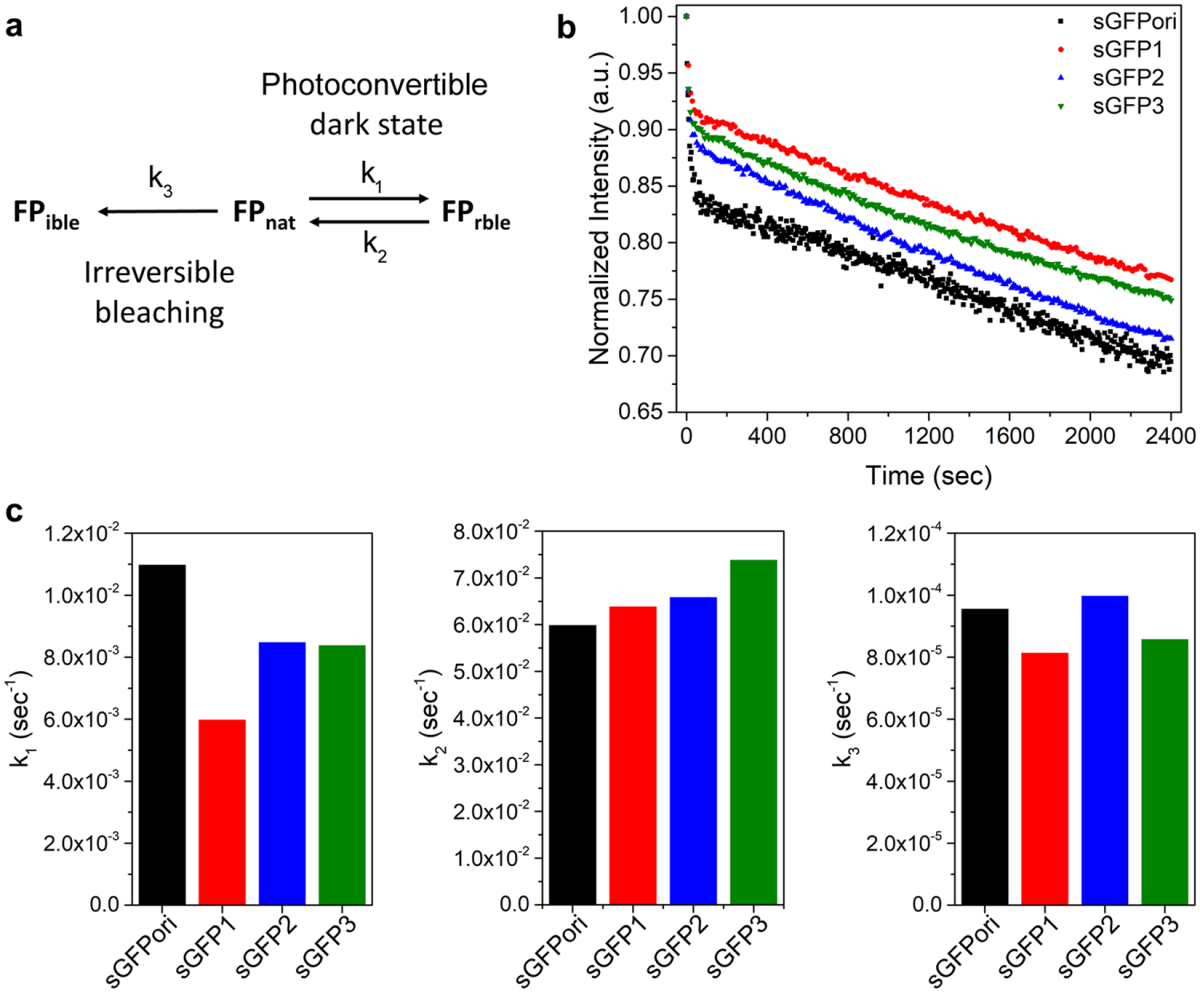
Photobleaching kinetic of complemented sGFP variants. (**a**) Model for irreversible photobleaching (FP_ible_) and reversible photoconvertible dark state reactions (FP_rble_) for a native GFP (FP_nat_). (**b**) Normalized photobleaching kinetics for complemented sGFPori, sGFP1, sGFP2 and sGFP3. (**c**) Comparison of the forward (k_1_, left panel) and backward (k_2_, middle panel) photoconvertible dark state rate constants and of the irreversible photobleaching rate constants (k_3_, right panel) for all complemented sGFP variants.

In addition to studying the quantum yield and extinction coefficient of the complemented sGFP variants, we also compared their ensemble photobleaching characteristics with that of complemented sGFPori under constant 488 nm excitation. Photobleaching kinetics in EGFPs generally involve two phases: (i) a photoconvertible dark state phase, characterized by a rapid but reversible decrease in fluorescence intensity, and (ii) an irreversible photobleaching phase characterized by a slower and continuous decrease in fluorescence due to photo-destruction of the chromophore^50^ (Fig. 2a and Supplementary Information). The forward and backward photoconvertible dark state rates (*k*_1_ and *k*_2_) as well as the irreversible photobleaching rate (*k*_3_), were determined by fitting the photobleaching kinetic of each complemented sGFPs with a set of differential equations (Fig. 2b and Supplementary Information). Among the complemented sGFPs, sGFPori shows the fastest *k*_1_ rate towards the photoconvertible state and the slowest backward rate from this dark state (*k*_2_) indicating that it is more prone to be trapped in a non-fluorescent dark state than other green variants (Fig. 2c). The V167T substitution in sGFP1 limits trapping in the dark state, mainly by decreasing the forward *k*_1_ rate towards the photoconvertible state. Compared to sGFP1, the additional S72A (sGFP2) or S72A/N149K substitutions (sGFP3) slightly increase the *k*_1_ rate toward the dark state but both variants still display ~25% slower entry into the photoconvertible state than sGFPori, as well as faster *k*_2_ exit rates from this non-fluorescent state (Fig. 2c). Changes in the amplitude of the initial fluorescence intensity due to equilibrium between the bright and dark state of the complemented sGFPs under our photobleaching kinetic conditions (*k*_1_/*k*_2_) were 18%, 9%, 13% and 11% for complemented sGFPori, sGFP1, sGFP2 and sGFP3 respectively. Concerning the photobleaching rates (*k*_3_), the V167I mutation in sGFP1 slows down irreversible photobleaching by 15% compared to sGFPori. However, the additional S72A substitution in sGFP2 cancels this effect, which is partially recovered by the S72A/N149K substitutions in sGFP3. Overall, complemented sGFPori shows the highest probability to be trapped in the photoconvertible dark state and photobleaches faster than complemented sGFP1 or sGFP3. Complemented sGFP2 is less prone to enter and stay in the photoconvertible dark state compared to sGFPori but photobleaches at a rate similar to that of sGFPori under continuous excitation.

### Folding kinetics of full-length FP variants

**Figure 3:**
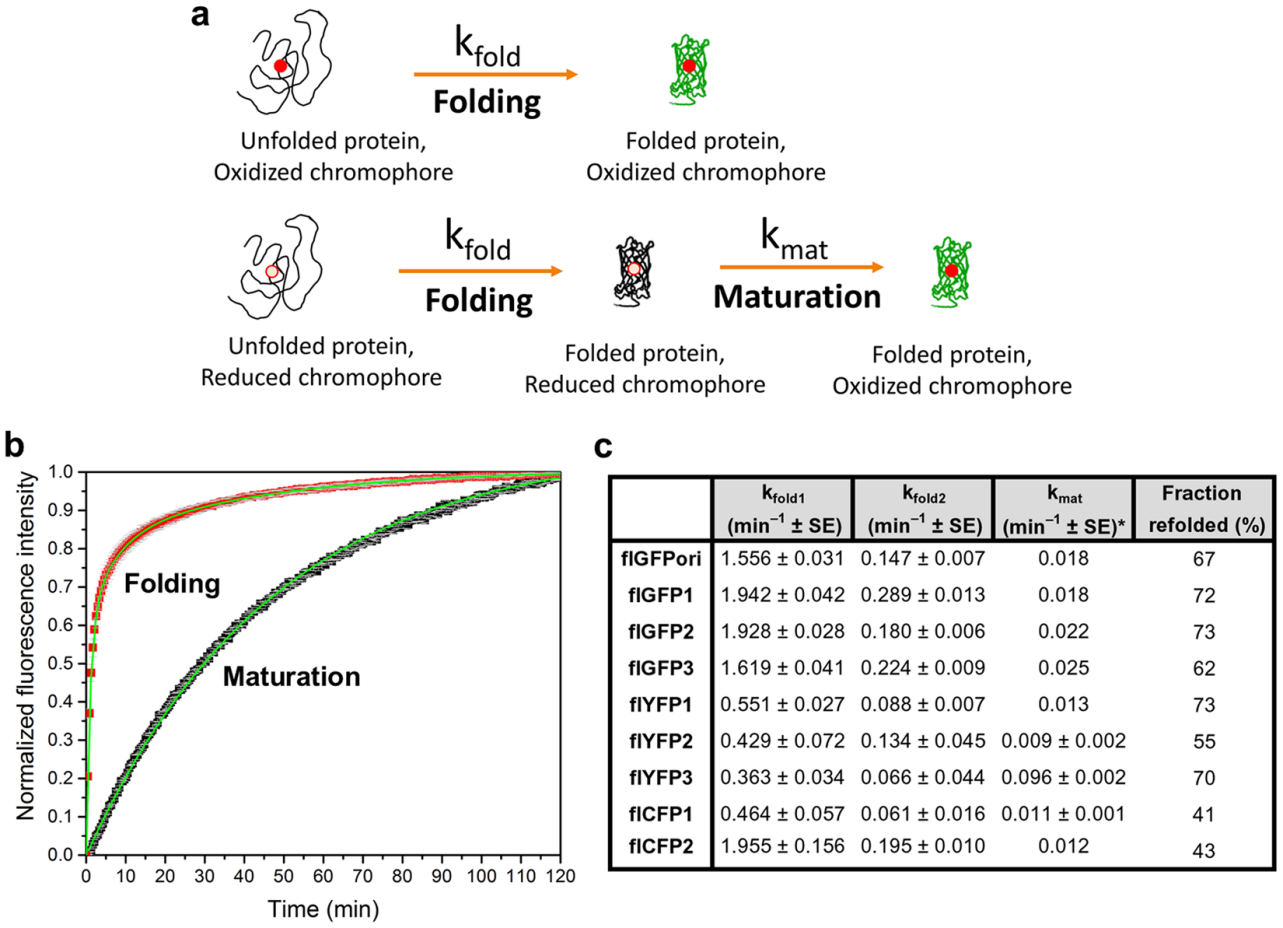
Folding and maturation of full-length FPs. (**a**) Schematic models of FP folding and maturation processes. (**b**) Example of flGFP2 folding (red) and maturation (black) kinetics with tri-exponential and single exponential fits (green lines), respectively. (**b**) Comparison of k_fold1_, k_fold2_, k_mat_, and refolding efficiencies between all the flFP variants. SE: standard error of the fit. *: SE lower than three decimals are not reported.

The folding rates of flFPs were determined by monitoring the fluorescence recovery of the chromophore after denaturation of the FP β-barrel in urea at 95 °C and dilution-induced refolding in denaturant-free buffer^51,52^ (Fig. 3a). This treatment induces a loss of the FP native structure and exposes the mature and chemically intact chromophore to the surrounding environment, leading to an initial quenching of fluorescence that recovers as FPs regain their tertiary structure in a denaturant-free buffer^53,54^. The folding kinetics of the all the flFP variants were assessed by fitting this fluorescence recovery with a tri-exponential function (Fig. 3b) in order to define three independent and first-order kinetic rate constants: *k*_*fold1*_ and *k*_*fold2*_, which correspond to the rate constants of two parallel folding pathways involving properly and improperly isomerized proline residues^53^, respectively and *k*_*mat*_, which corresponds to the rate constant of chromophore maturation for a small amount of misfolded and none-matured FPs in our purified samples. For flGFPori, the *k*_*fold1*_ and *k*_*fold2*_ folding rate constants are similar to those observed previously for urea-unfolded S65T-GFP^53^ (*k*_*fold1-flGFPori*_ of 1.556 min^−1^ *vs*. *k*_*fold1-S65TGFP*_ of 1.470 min^−1^ and *k*_*fold2-flGFPori*_ of 0.147 min^−1^ *vs*. *k*_*fold2-S65TGFP*_ of 0.146 min^−1^)^53^ and its refolding efficiency is 62% (Fig. 3c). These folding rates are also in good agreement with the multiphase refolding kinetic reported for the same flGFPori by Huang and Bystroff^5^, although our different denaturing and acquisition conditions did not allow us to detect a very fast flGFPori folding rate constant mentioned by these authors. We also detected a much slower rate constant (*k*_*mat*_, 0.018 min^−1^, amplitude typical 10–20% of the total kinetic recovery), which we assigned to the chromophore maturation as it systematically matched the maturation rate constants determined independently in reduced chromophore maturation kinetics (Fig. 3c). Both folding rate constants and folding efficiencies are improved by the substitutions V167T (flGFP1), V167T/S72A (flGFP2) and V167T/S72A/N149K (flGFP3) (Fig. 3c). The additional N149K substitution in flGFP3, however, results in a lower refolding efficiency compared to flGFPori, despite improvements in folding rates.

For the flYFP variants, the *k*_*fold1*_ rate constants are 0.551 min^−1^ for flYFP1, 0.429 min^−1^ for flYFP2 and 0.363 min^−1^ for flYFP3, all of which are faster than the previously reported folding rates for EYFP (0.24 min^−1^)^55^. These improvements in folding are likely the result of the folding reporter and superfolder mutations that were kept in all three flYFPs, with the additional T205S substitution in flYFP1 and flYFP2 participating to further improve folding kinetics compared to flYFP3. Both flYFP1 and flYFP3 display a relatively good refolding efficiency of 70–75%, but that of flYFP2 is only 55%.

For the flCFP variants, the observed fast folding rate constant of flCFP1 (0.464 min^−1^) is consistent with the previously reported folding rate of ECFP (0.66 min^−1^)^55^. Interestingly, the additional V167I mutation in flCFP2 dramatically improves both *k*_*fold1*_ (1.955 min^−1^) and *k*_*fold2*_ (0.195 min^−1^) compared to flCFP1. With respect to all the other flFP variants, the refolding efficiency of both flCFPs is low and below 50%.

### Chromophore maturation kinetic of full-length FP variants

In addition to the flFPs folding rates, we also determined the chromophore maturation rates of each variant using dithionite as a reducing agent during denaturation and measuring the rate-limited oxidation step of the chromophore^25,53^ upon dilution-induced refolding in denaturant-free and dithionite-free buffers (Fig. 3a). For each flFPs, the fluorescence recovery of the reduced chromophore was fitted by a single exponential function to extract the irreversible and first-order maturation rate constant k_mat_ (Fig. 3b). As seen in Fig. 3c, flGFPori matures with a rate constant of 0.018 min^−1^, equivalent to that of wild-type GFP^23^. While the V167T substitution in flGFP1 does not improve the maturation rate (k_mat_: 0.018 min^−1^), the additional S72A substitution in flGFP2 (k_mat_: 0.023 min^−1^) and S72A/N149K in flGFP3 (k_mat_: 0.025 min^−1^) both result in faster chromophore maturation, as expected^23^. These maturation rates, however, remain slower than previously reported for Emerald-GFP^23^ (k_mat_: 0.084 min^−1^) which matures nearly 3.5 time faster than flGFP2 or flGFP3. flYFP1 (k_mat_: 0.013 min^−1^) and flYFP2 (k_mat_: 0.009 min^−1^) are significantly slower maturing proteins than the flGFP variants (Fig. 3c), but flYFP3 displayed a very fast maturation with a rate constant of 0.096 min^−1^, which is a significant improvement compared to other YFPs such as EYFP (k_mat_: 0.043 min^−1^) or Venus (k_mat_: 0.025 min^−1^)^23^. This fast maturation of flYFP3 might be linked to the shorter and less polar T205A amino acid substitution which could favor easier conveyance of bulk solvent molecules and oxygen to the pre-cyclized chromophore as previously proposed for a T203V/S205A GFP variant^56^. The flCFP variants flCFP1 (k_mat_: 0.011 min^−1^) and flCFP2 (k_mat_: 0.012 min^−1^) have maturation rate constants similar to that previously reported for ECFP (k_mat_: 0.0096 min^−1^)^55^ and the V167I substitution in flCFP2 does not impact the maturation rate (Fig. 3c).

### Self-assembly process of split-FPs

**Figure 4:**
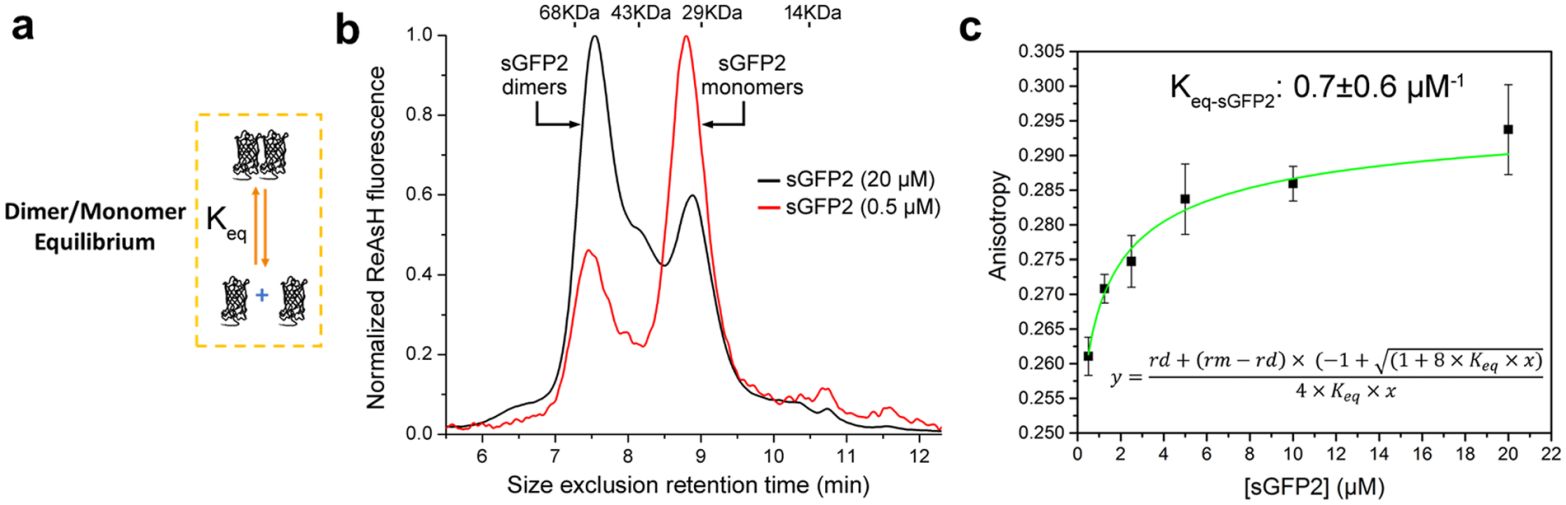
Concentration-dependent dimer-monomer exchanges in recombinant split-fluorescent proteins. (**a**) Schematic of dimer-monomer equilibrium in recombinantly produced sFPs. *K*_*eq*_ represents the equilibrium constant for dimer formation. (**b**) Size exclusion high-pressure liquid chromatography of non-complemented sGFP2 labeled with fluorescent ReAsH on an N-terminal tetracysteine tag. sGFP2 is mostly dimeric at 20 µM (apparent molecular weight of 61 KDa) but mostly monomeric at a lower concentration of 0.5 µM (apparent molecular weight of 31 KDa). The retention times of a set of calibrated molecular weight standards (68, 43, 29 and 14 KDa) are provided as reference. (**c**) Steady-state fluorescence anisotropy of ReAsH-labeled sGFP2 at different concentrations. The apparent equilibrium constant of dimer formation (*K*_*eq*_) is determined by fitting the anisotropy curve with the inset equation (green), which describes the ensemble anisotropy contributed by both dimer anisotropy (*rd*) and monomer anisotropy (*rm*) at each total sGFP2 concentration. Anisotropy values are presented as mean ± std from measurements in triplicate.

Having characterized the folding and maturation processes in flFPs, we then studied the assembly kinetics of sFPs with a complementary 11^th^ β-sheet M3 peptide produced synthetically and additionally determined their complementation-induced chromophore maturation rates. As a starting model, we first considered the fact that recombinantly produced sGFPori and all the sFP variants are mostly dimeric at high concentrations, as previously reported^3^, but that sFP monomers become the dominant fraction at low protein concentrations (Fig. 4a,b). This concentration-dependent dimer-monomer equilibrium of sFPs was further characterized by steady-state fluorescence anisotropy measurements using ReAsH, a fluorescent dye that rigidly labels a tetracysteine tag^57^ encoded at the N-terminus of each sFP. As shown in Fig. 4c for ReAsH-sGFP2, fluorescence anisotropy decreases as a function of decreasing total sFP concentrations, confirming the concentration-dependent dimer dissociation of sFPs. The apparent equilibrium constant of dimer formation (K_eq_) was determined by fitting this anisotropy curve with a model for dimer-monomer dissociation^58^ (Fig. 4c, inset equation). For sGFP2, the observed K_eq_ of dimer formation is 0.7 ± 0.6 μM^−1^, while that of sGFPori is comparable at 0.32 ± 0.2 μM^−1^.

**Figure 5:**
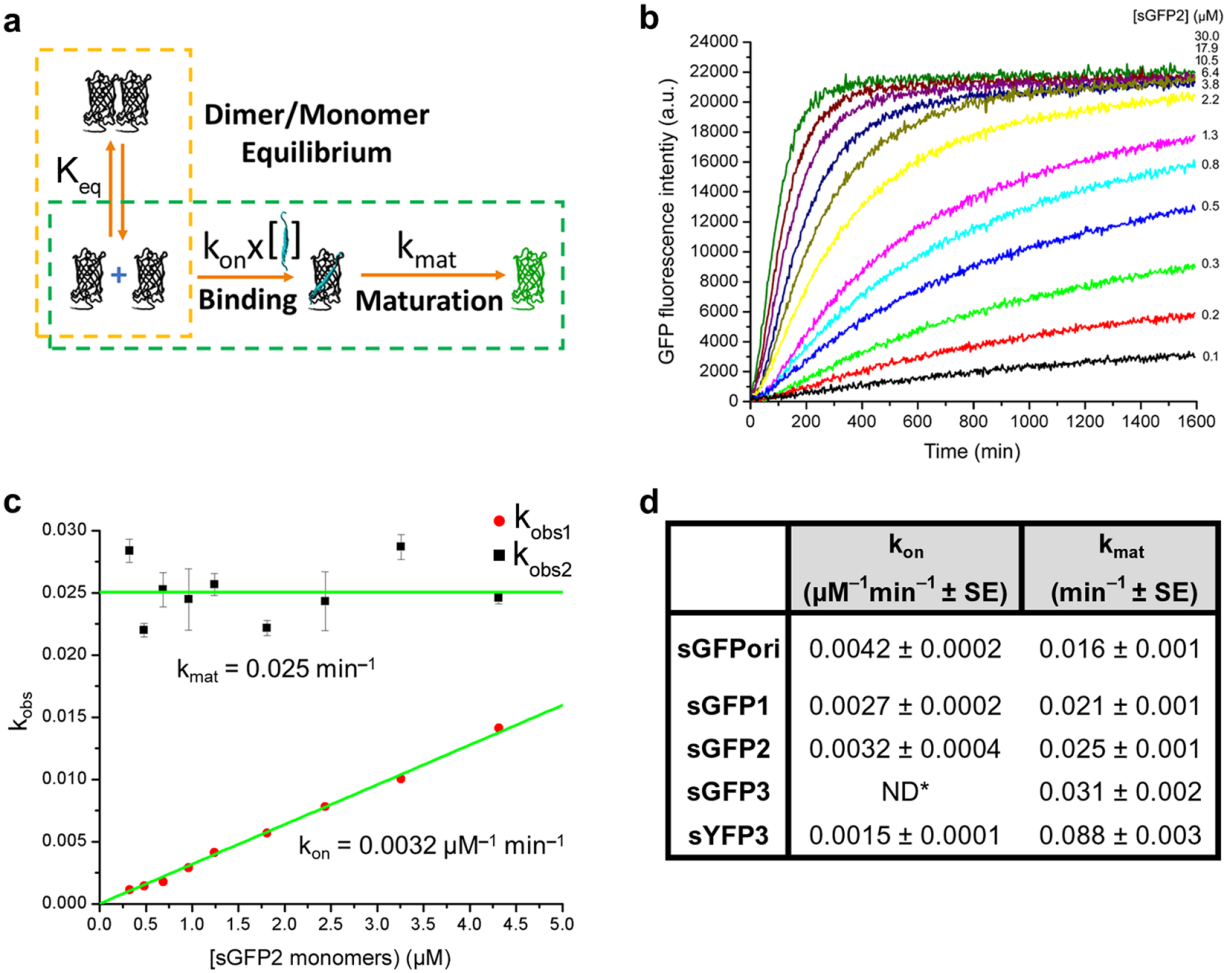
Assembly kinetics of split-fluorescent protein fragments. (**a**) Schematic of sFP complementation and maturation, including the sFP dimer-monomer equilibrium (K_eq_), an irreversible binding step of complementary M3 peptides to sFP monomers with rate constant k_on_ and an irreversible chromophore maturation step with rate constant k_mat_. (**b**) Example of pseudo-first order fluorescence kinetic curves for increasing concentrations of sGFP2 incubated with 0.1 µM of complementary M3 peptides. Only one replicate out of three performed is shown for clarity. Under these conditions, the dimer-monomer equilibrium (orange dashes in (**a**)) does not affect the binding and maturation reactions (green dashes in (**a**)), allowing the observed binding rate k_obs1_ and maturation rate k_obs2_ to be determined at each sGFP2 concentration with a bi-exponential fit. (**c**) Distribution and fit (green) of k_obs1_ and k_obs2_ as a function of monomeric sGFP2 concentration to define the rate constants k_on_ and k_mat_, respectively. k_obs_ values are presented as mean ± std from measurements in triplicate. (**d**) Comparison of k_on_ and k_mat_ rate constants for some complemented sFPs. ND*: Not determined because sGFP3 exists as complexes bigger than dimers or monomers when expressed recombinantly. SE: standard error of the fit.

Next, we considered a simple sFP complementation model that involves a first irreversible binding step of complementary M3 peptides only to monomeric sFPs, followed by a second irreversible chromophore maturation step (Fig. 5a). Indeed, as previously reported for sGFPori^3,14^ and other FP-based bimolecular fluorescence complementation systems^59^, binding of complementary fragments and FP reassembly are irreversible and complemented fluorescent sGFPori are always monomeric. To assess the validity of this model, we studied the fluorescence kinetic of sFP assembly with M3 peptide under pseudo-first order conditions, where increasing concentrations of sFP are titrated on a small but constant concentration of M3 peptide (Fig. 5b). Fluorescence kinetic curves at different sFP concentrations were fitted with a bi-exponential function to determine the apparent rates k_obs1_ and k_obs2_ that define the irreversible binding and chromophore maturation steps, respectively. As shown for sGFP2, the distribution of k_obs1_ is linearly dependent on the concentration of sGFP2 monomers determined using the equilibrium constant of sGFP2 dimer formation, but displayed a non-linear dependence on sGFP2 dimer concentrations and on total sGFP2 concentrations (dimer and monomers), consistent with a binding of complementary M3 peptides to monomeric sGFP2 only (Fig. 5c). k_obs2_, however, remains constant at all sGFP2 concentrations, as expected for an independent chromophore maturation step that follows the assembly of sGFP2 monomer-M3 peptide complexes (Fig. 5c). By fitting the distribution of k_obs1_ with a simple linear function we determined a k_on_ binding rate constant of 0.0032 µM^−1^ min^−1^ with a y intercept at 0, fully consistent with the irreversible binding between sGFP2 monomers and M3 peptides. Fitting the distribution of k_obs2_ with a constant resulted in the determination of the complemented sGFP2 maturation rate constant k_mat_ at 0.025 min^−1^, in good agreement with the maturation rate of flGFP2 determined independently (0.022 min^−1^, Fig. 3c). This indicates that the maturation rates of sFPs and flFPs are very similar. The k_on_ binding rate constants determined for some of the other complemented sFP variants are within two-folds of that measured for sGFP2, with sGFPori having a slightly faster binding rate constant than sGFP2 at 0.0042 µM^−1^ min^−1^ (Fig. 5d). The maturation rate constants measured from fluorescence complementation kinetics for these sFP variants are again in good agreement with those determined independently for the corresponding flFP variants (Fig. 5d). We note that the measured k_on_ values for sFPs are 3–5 orders of magnitude slower than the rates expected for diffusion-limited reactions^60^. This suggests that k_on_ represents a slow and rate-limiting steric fit process between the complementary sFP and M3 fragments that takes place after diffusion and interaction, both of which are nonetheless required for the two fragments to interact. These kinetic assays also indicate that, in this asymmetric sFP fragment system, complementation follows a conformational selection process, whereby the larger split-FP fragments exist in a dimer-monomer equilibrium and only monomers are competent for a slow but irreversible binding to the small M3 peptide fragment, followed by maturation of the FP chromophore at a rate similar to that of flFPs.

### Live cells expression, fluorescence imaging and oligomerization of split-FP fusion protein variants

**Figure 6:**
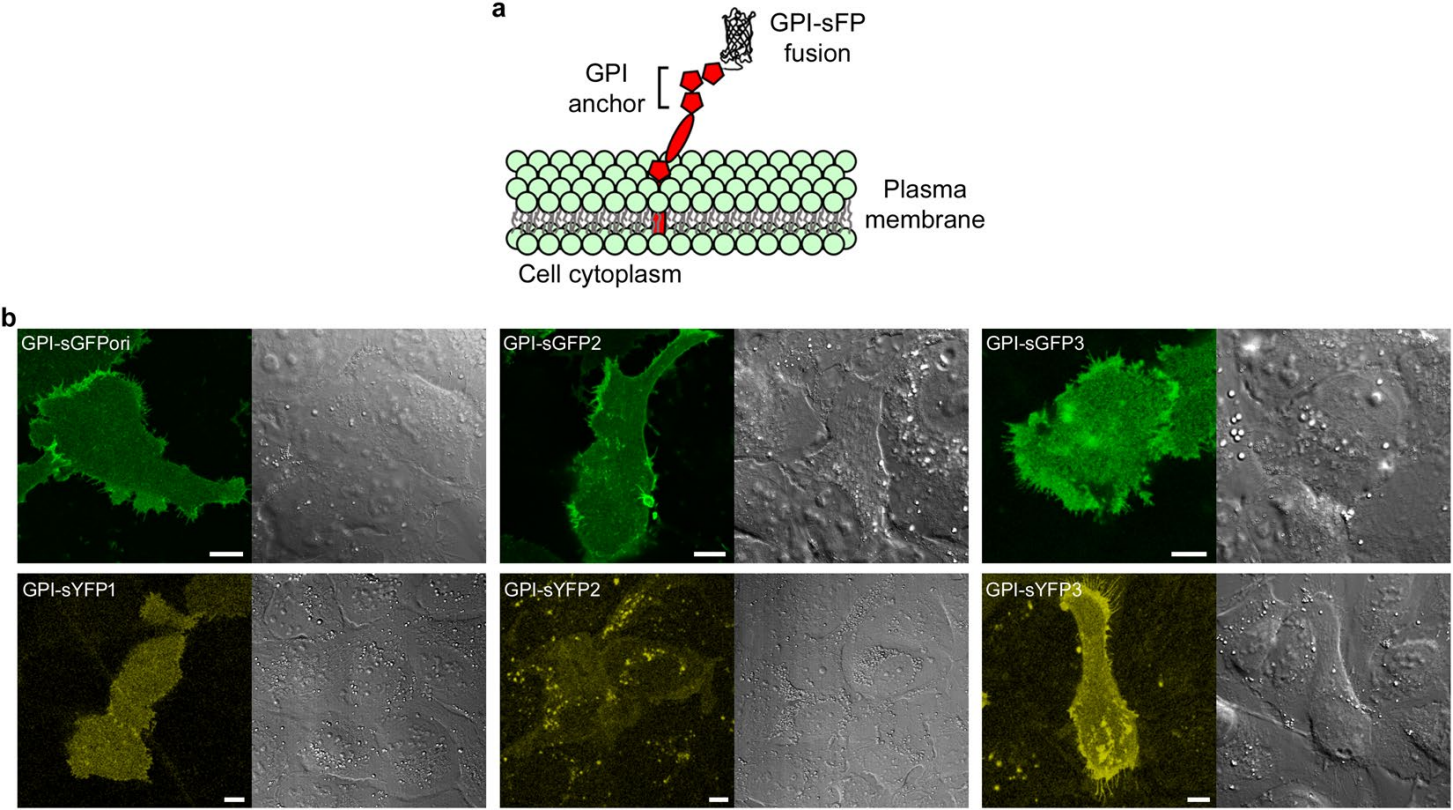
Live cell confocal imaging of complemented GPI-anchored split-FP fusions. (**a**) Schematic representation of GPI-sFP fusions expressed at the outer leaflet of the plasma membrane in U2OS cells. (**b**) Fluorescence confocal images of different complemented GPI-sFPs at the cell ventral plasma membrane (left) and corresponding differential interference contrast images. Scale bars: 10 μm.

We then selected some of the sFP variants to test their expression as plasma membrane fusion proteins in mammalian cells and compared their fluorescence complementation with that of sGFPori using exogenous synthetic M3 peptides and CALM imaging^14^ in live cells. Amongst the sGFPs, both sGFP2 and sGFP3, which respectively have 16 min and 21 minutes faster chromophore maturation half-times than sGFPori, were fused to the glycosylphosphatidyl inositol (GPI) anchoring domain of the plasma membrane protein CD14 and transiently expressed in U2OS cells (Fig. 6a). All the sYFP and sCFP variants were also expressed as GPI-fusions. As seen in fluorescence confocal images taken at the ventral plasma membrane of transfected cells, incubation with M3 peptides results in an effective complementation and fluorescence activation of all the GPI-sGFP and GPI-sYFP variants, although the detection of GPI-sYFP2 is difficult because of its low brightness (Fig. 6b**)**. As verified by cross-sectional confocal imaging, fluorescence activation specifically takes place at the cell plasma membrane of transfected cells (Supplementary Fig. S2). The rapid lateral diffusion of individual activated sFPs observed by total internal reflection fluorescence (TIRF) microscopy additionally indicates that the GPI-fusions are properly targeted to the cell surface (Supplementary Video V1). However, no cell surface fluorescence complementation could be observed for either GPI-sCFP1 or GPI-sCFP2. Indeed, anti-CFP immunostaining of transfected cells revealed that the sCFP fusions are not translocated to the plasma membrane but are retained in the endoplasmic reticulum (Supplementary Fig. S3), suggesting a misfolding of sCFP fusions when expressed in mammalian cells at 37 °C. This observation is consistent with the reduced *in vitro* refolding efficiency of flCFPs compared to other color variants (Fig. 3c).

The effective fluorescence complementation of different GPI-sGFPs and GPI-sYFPs in live cells suggests that there is a substantial fraction of monomeric fusion proteins at the cell surface, since, as determined biochemically, only sFP monomers are competent for fluorescence activation upon binding complementary M3 peptides (Fig. 5). To assess the possible additional presence of dimeric GPI-sFP fusions at the cell plasma membrane, we employed the membrane impermeable and amine-reactive bifunctional cross-linker bis(sulfosuccinimidyl)suberate (BS3), whose small 1.2 nm size^61^ allows the cross-linking of proteins that are in very close proximity within the cell membrane, including dimers^62^. When GPI-sGFP2 expressed in U2OS cells was cross-linked with BS3, extracted and analyzed by denaturing SDS-page electrophoresis and immunoblotting, no enrichment of GPI-sGFP2 dimers was observed, indicating that GPI-sFP fusions are only monomeric at the cell surface (Supplementary Fig. S4 and Supplementary Information). This is in contrast with the dimer-monomer equilibria observed for recombinantly expressed and purified sFPs. It indicates that the complementation efficiency and fluorescence activation of sFP fusion proteins in cells is only dependent on the concentration of M3 peptides and on the binding (k_on_) and maturation (k_mat_) rate constants of each sFP, provided that they undergo correct cellular expression and folding.

### Photophysical properties of individual complemented sGFPori and sGFP2 fusion proteins in cells

**Figure 7:**
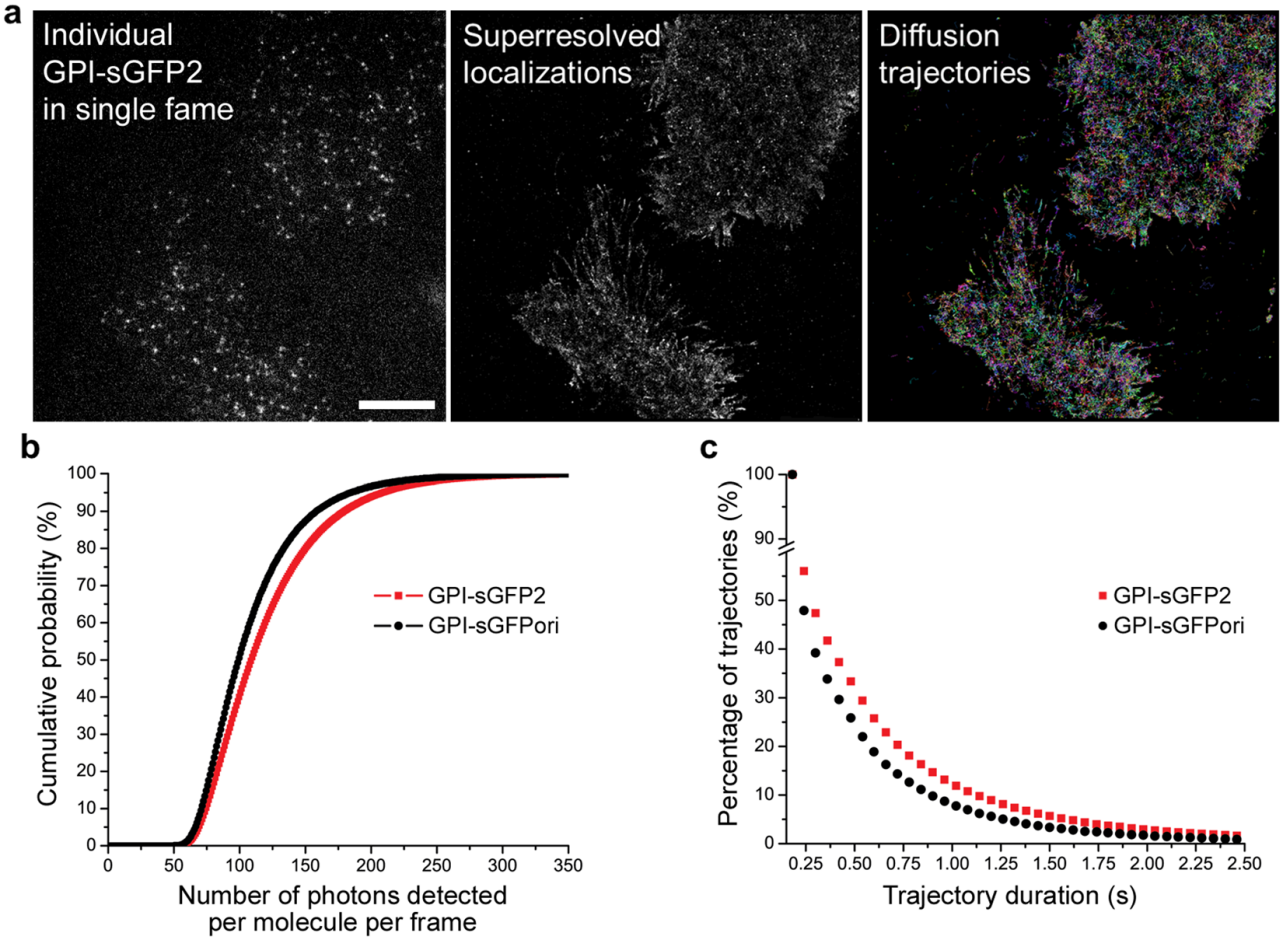
Single molecule imaging and tracking of complemented GPI-anchored split-GFP protein fusions in cells. (**a**) Single frame TIRF image (left), full acquisition 2D-Gaussian super-resolved localizations (center) and reconstructed diffusion trajectories (right) for individual complemented GPI-sGFP2 at the plasma membrane of U2OS cells. Scale bar: 10 μm. (**b**) Comparison of individual molecule brightness for complemented GPI-sGFPori and GPI-sGFP2 at the plasma membrane. (**c**) Comparison of trajectory durations after single molecule tracking of GPI-sGFPori and GPI-sGFP2 at the cell plasma membrane.

We then tested and compared the properties of complemented sGFPori and sGFP2 for single molecule CALM imaging and tracking in live cells^14^. Cells expressing GPI-sGFPori or GPI-sGFP2 were imaged by TIRF after incubation with the complementary M3 peptide fragment to compare brightness and photostability between both fusions at the cell surface. As shown for GPI-sGFP2 expressing cells, CALM imaging by TIRF excitation at 488 nm results in the appearance of individual complemented GPI-sGFP2 fusion proteins at 520 nm after addition of M3 peptides (Fig. 7a). Individual GPI-sGFP2 appearing at the plasma membrane were localized by two-dimensional Gaussian fitting of their diffraction-limited point-spread function and their diffusion trajectories were reconstructed by linking the localized position of each molecule from frame to frame (Fig. 7a).

When imaged under the same conditions, individual complemented GPI-sGFP2 display significantly brighter fluorescent signals than GPI-sGFPori (Fig. 7b, Kolmogorov-Smirnov test: P < 0.05) with a mean number of photons per molecule and per frame of 121 ± 46 photons for GPI-sGFP2 and 109 ± 40 photons for GPI-sGFPori, under our imaging settings. This 11% percent increase in emitted photons for individual GPI-sGFP2 compared to GPI-sGFPori is consistent with the fact that, as determined by ensemble *in vitro* measurements (Table 1), sGFP2 is 15% brighter than sGFPori due to its higher quantum yield. In addition, there are more complemented GPI-sGFP2 displaying long trajectory durations compared to GPI-sGFPori (Fig. 7c). While, this might suggest that GPI-sGFP2 is more photostable than GPI-sGFPori, such a hypothesis is not reflected by ensemble photobleaching measurements where both complemented sGFPs display similar irreversible photobleaching properties (Fig. 2b,c). A more likely explanation for the improved single molecule tracking length with sGFP2 is that it is less prone to be trapped in the photoconvertible dark state compared to sGFPori, which reduces the probability of blinking and temporary loss of fluorescence signal, thus favoring consecutive frame tracking of individual GPI-fusions over longer periods of times. Combined with its overall faster maturation rate than sGFPori, the increased brightness and reduced dark state trapping of sGFP2 therefore provide improvements over sGFPori when it is used as a protein fusion tag for single molecule tracking by CALM imaging in live cells.

## Discussion

We have developed better folding and faster maturing sGFP variants of sGFPori using specific point mutations and have characterized its sYFP and sCFP spectral variants. A comparison between the flFPs and their complemented split forms indicates that they exhibit identical spectral properties, but that reconstructed sFPs have slightly reduced quantum yield and fluorescence lifetimes due to a less sturdy β-barrel structure. The various Emerald-GFP amino acid substitutions implemented in flGFPori/sGFPori resulted in green variants with improved quantum yield and brightness except for complemented sGFP3 where the surface N149K substitution impacts protein stability and quantum yield in the split form of flGFP3. These substitutions also significantly reduce the propensity of complemented sGFPori to be trapped in a non-fluorescent reversible dark state and lead to faster folding of flGFPs and faster chromophore maturation for both flGFPs and re-assembled sGFPs. Amongst the yellow variants, flYFP1/sYFP1 are the brightest YFPs with faster folding but reduced brightness and maturation time compared to EYFP. Amongst the cyan variants, flCFP2/sCFP2 are the brightest CFPs with faster folding, equivalent maturation time but reduced brightness compared to ECFP. We additionally showed that the large split-FP fragment exists in a concentration-dependent monomer-dimer equilibrium when expressed as a non-fusion protein *in vitro* and that only monomeric split-FPs are competent for fluorescence complementation upon binding M3 peptides. Compared to diffusion limited reactions, this binding step is slow but irreversible and it is followed by the maturation of the chromophore at a rate similar to that of flFPs. We note that when sFPs are used as non-fusion recombinant proteins co-expressed in cells with proteins fused to M3 complementary peptides^13,63^, this dimer-monomer equilibrium does not appear to affect fluorescence detection, although it might potentially impact the complementation efficiency and its kinetics if expression ratios between M3-protein fusions and recombinant sFPs are not well controlled. In contrast, when the large sFP fragment is used as a protein fusion in cells, it behaves as a monomer that can be controllably activated with synthetic M3 fragments for ensemble or single molecule fluorescence microscopy, as demonstrated here for various GPI-sFP fusions. A comparison of the photophysical properties of individual GPI-sGFPori and GPI-sGFP2 fusion proteins in live cells indicated that sGFP2 is brighter and allows longer single molecule tracking and trajectory reconstructions than sGFPori, consistent with its higher quantum yield and the lower propensity to be trapped in a light-induced photoconvertible dark state compared to sGFPori. Together with its increased maturation rate, sGFP2 provides a good balance between M3 peptide binding rate, brightness and photostability for fluorescence imaging applications and for the controlled assembly of nanomaterials and protein-based super-structures using complementary sFP fragments as scaffolds.

## Methods

### Expression and purification of recombinant full-length and split-FPs

A Quickchange lightning site-directed mutagenesis kit (Agilent Technologies) and appropriate template primers were used to make site-directed mutations and design the spectral variants of sGFPori and flGFPori. All the mutations in the described variants were verified by DNA sequencing. DH5α competent cells were used to expand all plasmids. Plasmids encoding flFPs and sFPs with a N-terminal 6xHis-tag, a GSS linker sequence, a thrombin cleavage site, a tetracysteine motif and a flexible GGSGG linker domain were transformed in a BL21(DE3) *E*. *coli* strain for protein expression. Overnight starter culture prepared with a single transformed *E*. *coli* colony was inoculated into 1 L LB (35 μg/ml kanamycin) and the culture was incubated in a shaker at 37 °C until OD_600_ reached ~0.6. The culture was cooled down at room temperature (RT) for expression induction with 1 mM IPTG and incubated overnight at 20 °C. Cells were then harvested at 4000 g for 30 min at 4 °C and the cell pellet was washed with ice cold PBS at 4000 g for 30 min at 4 °C and re-suspended in TN/imidazole buffer (100 mM Tris-HCl, 150 mM NaCl, 10 mM imidazole, pH 8.0). 1× HALT protease inhibitor, 0.5 mM TCEP, 5 μl benzonase nuclease/g of cell pellet and 5 ml 1× bugbuster/g of cell pellet (EMD Millipore) were added and incubated for 30 min at RT for cell lysis. Samples were centrifuged at 16000 g for 15 min at 4 °C and the supernatant containing flFPs or sFPs was collected. Ni-resin beads were used to purify each FPs. TN buffer with 10 mM imidazole and 150 mM imidazole were used as wash buffer and elution buffer, respectively. After purification, samples were dialyzed twice against 1 L TN buffer (100 mM Tris-HCl, 150 mM NaCl, pH 8.0) at 4 °C for 1 hour then overnight, in order to remove excess imidazole. As determined by SDS page electrophoresis, flFPs and sFPs sample were >95% pure. A BCA protein assay was used to determine the respective concentrations of each FP. The 6xHistag was then removed by thrombin cleavage (15 U/mg protein) for 20 min at RT in the presence of 1 mM TCEP. *p*-aminobenzamidine beads were used to eliminate residual thrombin after cleavage and the proteins were further dialyzed twice against 1 L TN buffer at 4 °C for 1 hour then overnight to remove TCEP. flFPs and sFPs were frozen in TN + 10% glycerol using liquid nitrogen and stored at −80 °C. As determined by size-exclusion HPLC, all the flFPs were more than 90% monomeric at 250 µM concentration, while all the sFPs displayed a concentration dependent dimer:monomer equilibrium, except for sGFP3 which displayed additional complexes with molecular weights higher than expected for dimers and monomers.

### Spectral acquisitions and photobleaching kinetics of FPs

A Varian Cary® 50 UV/Vis spectrometer and a Horiba Nanolog spectrofluorometer were used to acquire absorption and emission spectra of all FPs and for photobleaching kinetics. Purified flFP variants were diluted at 5 μM in TNG buffer (100 mM Tris-HCl, 150 mM NaCl, 10% glycerol, pH 8.0). Fluorescence complementation of sFPs (20 μM) was done by incubation with a synthetic M3 peptide (200 μM, GSGGGSTSRDHMVLHEYVNAAGIT) for 12 hours in TNG buffer. For photobleaching kinetics, complemented sGFP variants in TNG buffer were constantly excited at 488 ± 1 nm and fluorescence emission was collected at 530 ± 1 nm for 40 minutes at RT. Photobleaching kinetic data were analyzed using Matlab by least-squares fitting the fluorescence decay curves with the solution to a set of differential equations describing the photoreversible and irreversible photobleaching processes of GFPs (Supplementary Information).

### Extinction coefficients and quantum yields

Molar extinction coefficients (*ε*) were determined using Beer-Lambert law: A = *ε* × *C* × *l* where *A* is the absorbance, *C* is the concentration of the fluorophore, and *l* is the length of the light path through the FP sample. Quantum yields ($\Phi$) were calculated using a fluorescein standard (Sigma) with a quantum yield of $\Phi$ = 0.93 in 0.1 M NaOH. The absorption factors of the fluorescein standard (*f*_*st*_) and the FP samples (*f*_*x*_) were calculated using measured absorbance (*A*) at specific excitation wavelength (*λ*_*ex*_, 460 nm for GFPs, 440 nm for CFPs, 475 nm for YFPs) using:

1 $$f=1-{10}^{-A({\lambda }_{ex})}$$

The relative integral photon fluxes emitted from the fluorescein standard (*F*_*st*_) and the FP samples (*F*_*x*_) were calculated based on the spectrally corrected and blank-corrected spectrum of each sample (I_C_) using:

2 $$F=\mathop{\int }\limits_{{\lambda }_{em}}{I}_{C}\times {\lambda }_{em}d{\lambda }_{em}$$

The fluorescence quantum yields were then calculated using:

3 $${\Phi }_{f,x}={\Phi }_{f,st}\times \frac{{F}_{x}}{{F}_{st}}\times \frac{{f}_{st}}{{f}_{x}}\times \frac{{n}_{x}^{2}({\lambda }_{em})}{{n}_{st}^{2}({\lambda }_{em})}$$

where *F* is the emitted relative integral photon flux, *f* is the absorption factor, *n* is the refractive index, *Φ*_*f*,*x*_ is the quantum yield of the sample and *Φ*_*f*,*st*_ is the quantum yield of the fluorescein standard. The *ε* and *Φ* values of each protein were used to calculate the brightness (*ε* × *Φ*).

### Fluorescence lifetime measurements

Fluorescence lifetime measurements were performed by two-photon frequency domain on a Zeiss LSM 780 inverted microscope equipped with 40X water immersion objective (NA 1.1) and hybrid photodetectors (Hamamatsu). flFPs and complemented sFPs in TNG buffer were excited by a two-photon laser at 870 nm (5–10 mW) (Chameleon, Coherent) with a 150-fs pulse bandwidth and at 80 MHz repetition rate. A 537DF26 nm bandpass emission filter was used for GFPs/YFPs and a 483DF32 nm bandpass emission filter was used for CFPs. Acquisition times on the hybrid photodetectors were adjusted to achieve an average of 100 counts/pixel. The lifetime values were determined by least-square fitting the fluorescence decay curves with the following one or two-component exponential fits using an ISS VistaVision software (version 4.1):

4 $$y=A{e}^{(-t/{\tau }_{1})}$$

or

5 $$y={A}_{1}{e}^{(-t/{\tau }_{1})}+\,{A}_{2}{e}^{(-t/{\tau }_{2})}$$

### Refolding and maturation kinetics of full-length FPs

Refolding kinetics were done by first boiling 50 μM of each flFPs at 95 °C for 10 minutes in TNG with 8 M urea, 1 mM DTT and 0.2 mM EDT added to reduce potential disulfide bonds. Denatured samples prepared in triplicate were then diluted by 100-fold in TNG on a 96-well microplate to induce refolding. A Biotek Synergy H4 microplate reader equipped with a xenon lamp, and appropriate excitation/emission filters (452DF17/480DF10 nm for flCFPs, 485DF20/528DF20 nm for flGFPs or 500DF13/536DF10 nm for flYFPs) were used to acquire the refolding kinetics in 25 seconds increments for 2 hours at 25 °C. Microplate wells containing TNG buffer and 50 μM of non-denatured flFP variants were used for buffer background, photobleaching corrections and assessments of refolding efficiency. Refolding kinetics were analyzed with the following tri-exponential function using Origin 2016 as a software:

6 $$y={A}_{1}(1-{e}^{(-{k}_{fold1}x)})+\,{A}_{2}(1-{e}^{(-{k}_{fold2}x)})+\,{A}_{3}(1-{e}^{(-{k}_{mat}x)})$$

Maturation kinetic measurements were done by first denaturing 5 μM of each flFP variants at 95 °C for 10 minutes in TNG with 8 M urea, 1 mM DTT, 0.2 mM EDT and 5 mM dithionite to reduce the chromophore. The chromophore maturation was triggered by a 100-fold dilution of triplicate FP samples in TNG buffer and data were acquired on the Biotek Synergy H4 microplate reader as described above. Full-length FP samples were prepared without urea, dithionite or boiling for photobleaching corrections. Maturation kinetic curves were fitted by the following single exponential function using Origin 2016 as a software:

7 $$y=A(1-{e}^{(-{k}_{mat}x)})$$

### Self-assembly kinetic of split-FPs

Fluorescence complementation kinetics were performed in triplicate by incubating 0.1 μM of M3 peptide with various sFP concentrations (0.1–30 μM of total sFP dimers and monomers) in TN buffer pH 8.0 with 1 mM DTT, 5 mM EDTA and 0.05% CHAPS. Fluorescence signals were acquired every 3 minutes over a 14 hour period and at 25 °C on a Biotek SynergyH4 microplate fluorescence reader equipped with appropriate excitation and emission filters. Samples without M3 peptides were used for buffer background correction and a 0.1 μM solution of corresponding flFP was used for long-term photobleaching corrections. Kinetic curves were fitted with the following bi-exponential function using Origin 2016 as software:

8 $$y={A}_{1}(1-{e}^{(-{k}_{obs1}x)})+\,{A}_{2}(1-{e}^{(-{k}_{obs2}x)})$$

### Anisotropy measurements

For anisotropy measurements, sFPs at 40 μM were labeled with a 1:2 molar ratio of sFP to ReAsH dye in the presence of 5 mM TCEP and 2 mM BME for 1.5 hour at RT. Excess ReAsH was removed using a Sephadex gel filtration G-10 spin column (Harvard Apparatus). A Biotek SynergyH4 microplate reader equipped with vertical and horizontal polarization filters, a 540DF25 excitation filter and a 620DF40 emission filter was used to measure the steady state anisotropy values of ReAsH-sFPs at different dilutions (20, 10, 5, 2.5, 1.25 and 0.5 μM) after equilibrium at RT and in triplicate. Fluorescence anisotropy values were calculated as follows:

9 $$A=\frac{{I}_{\Vert }-G{I}_{\perp }}{{I}_{\Vert }+2G{I}_{\perp }}$$

where A is the fluorescent anisotropy value, *I*_ǁ_ is the parallel polarization intensity, I_⊥_ is the perpendicular polarization intensity, and G is the sensitivity correction factor of the instrument for the two detection modes. The equilibrium constants of dimer formation (K_eq_) were determined by least-squares fitting the anisotropy as a function of sFP concentration with the equation of Fig. 4c, using Origin 2016 as a software.

### Cell lines, cell labeling and confocal imaging

U2OS cells were maintained in DMEM (Lonza) supplemented with 10% fetal bovine serum (FBS, Gibco) in a humidified incubator at 37 °C, supplied with 5% CO2. A humanized cDNA encoding sGFPori fused to the GPI-anchoring domain of CD14^14^ was used as template to generate different GPI-sFP variants by site-directed mutagenesis (Quickchange, Agilent Technologies) with appropriate primers. All the constructs were verified by DNA sequencing. Cells transiently transfected with the different GPI-sFP fusions (XtremeGENE HP, Roche) were imaged after incubation with 37 μM of M3 peptide in the DMEM + 10% FBS culture media for 12 hours. Cells were briefly rinsed with HBSS buffer (Corning) and imaged in the same buffer at 37 °C. Confocal fluorescence images were acquired on a Nikon C2 inverted confocal microscope equipped with a 488 nm excitation laser and a 525DF50 nm emission filter for imaging complemented GPI-sGFP variants and with a 515 nm excitation laser and a 542DF27 nm emission filter for imaging complemented GPI-sYFP variants.

### Single molecule imaging and tracking

For single molecule imaging and tracking by CALM, U2OS cells were transiently transfected for 48 hours with plasmids encoding GPI-sGFPori or GPI-sGFP2. Cells were rinsed 3× with 37 °C HBSS (Corning), and imaged by TIRF microscopy in HBSS buffer after the addition of 45 μM M3 peptide. Total internal reflection fluorescence (TIRF) imaging was performed on a Nikon Eclipse Ti-E microscope equipped with a 100× 1.49 NA objective (Nikon), a iXon EMCCD camera (Andor), a laser line at 488 nm (Agilent), a multiband pass ZET 405/488/561/647× excitation filter (Chroma), a quad-band ZT405/488/561/647 dichroic mirror, and a 525/50 nm bandpass emission filter (Semrock). Particle analysis was performed using SlimFast, a single-particle detection and tracking software written in MATLAB that uses multiple-target tracing algorithms^64^. Localizations were performed on individual molecules by 2D Gaussian fitting of the point-spread function of each complemented GPI-sGFP in each frame. Diffusion trajectories were built by linking individual localized events from frame to frame, taking into account local particle densities. Trajectories with at least 3 steps were kept for analysis. Statistics for photon counts were performed on 250,000–350,000 single molecule localizations in 6–7 cells for each condition. Statistics for trajectory durations were performed on 30,000–40,000 trajectories tracked in 6–7 cells for each condition.

### Data availability

The datasets generated and/or analyzed during the current study are available from the corresponding author on reasonable request.

## Electronic supplementary material

Supplementary Information

Supplementary Video 1
