# Author Correction: Characterization of Split Fluorescent Protein Variants and Quantitative Analyses of Their Self-Assembly Process

**DOI:** 10.1038/s41598-019-55623-8

**Published:** 2019-12-06

**Authors:** Tuğba Köker, Anthony Fernandez, Fabien Pinaud

**Affiliations:** 1 Department of Biological Sciences, University of Southern California, 1050 Child Way, Los Angeles, 90089 California USA; 2 Department of Chemistry, University of Southern California, 1050 Child Way, Los Angeles, 90089 California USA; 3 Department of Physics and Astronomy, University of Southern California, 1050 Child Way, Los Angeles, 90089 California USA

Correction to: *Scientific Reports* 10.1038/s41598-018-23625-7, published online 28 March 2018

This Article contains errors in Table 1. E124V was incorrectly included in the ‘Substitutions’ column for flCFP2 and sCFP2. The correct Table 1 appears below.

**Table 1:** Fluorescence properties of full-length and split fluorescent protein variants. ε: Molar extinction coefficient, Φ: Quantum yield, ψ: Additional substitutions in flGFPori, δ: Additional substitutions in sGFPori, τ_1_ & τ_2_: Fluorescence lifetimes 1 & 2 for two-photon 870 nm excitation, A_1_ & A_2_: Fractions of fluorescence lifetimes 1 & 2.

	Exc. λ (nm)	Em. λ (nm)	ε(M^−1^ cm^−1^)	Φ	Brightness	τ_1_ (ns)	A_1_ (%)	τ_2_ (ns)	A_2_ (%)	Substitutions
**flGFPori**	485	507	37,700	0.66	24,880	2.46	100	—	—	S30R, N39I, F64L, S65T, F99S, T105K, E111V, I128T, Y145F, M153T, V163A, K166T, I167V, I171V, S205T, A206V, K221H, F223Y, T225N
**flGFP1**	485	507	33,800	0.75	25,350	2.5	100	—	—	^ψ^ V167T
**flGFP2**	491	510	38,200	0.77	29,410	2.77	100	—	—	^ψ^ V167T, S72A
**flGFP3**	482	508	39,900	0.68	27,130	2.67	100	—	—	^ψ^ V167T, S72A, N149K
**flYFP1**	511	522	68,300	0.61	41,660	2.76	100	—	—	^ψ^ T65G, T203Y, T205S
**flYFP2**	509	522	38,200	0.54	20,630	1.13	29	3.14	71	^ψ^ T65L, T203Y, T205S
**flYFP3**	515	526	37,100	0.51	18,920	0.72	37	3.59	63	^ψ^ T203Y, T205A
**flCFP1**	435	476	20,400	0.41	8,360	0.98	62	2.57	38	^ψ^ D19E, D21E, Y66W, E124V, H148D, T205S
**flCFP2**	436	476	21,300	0.42	8,950	0.97	27	2.76	73	^ψ^ D19E, D21E, Y66W, H148D, V167I, T205S
**sGFPori**	485	508	37,700	0.59	22,240	2.29	100	—	—	S30R, N39I, F64L, S65T, F99S, T105K, E111V, I128T, Y145F, M153T, V163A, K166T, I167V, I171V, S205T, A206V
**sGFP1**	485	508	33,800	0.62	20,960	2.34	100	—	—	^δ^ V167T
**sGFP2**	491	510	38,200	0.67	25,730	2.64	100	—	—	^δ^ V167T, S72A
**sGFP3**	480	508	39,900	0.29	11,600	2.5	100	—	—	^δ^ V167T, S72A, N149K
**sYFP1**	510	523	68,300	0.44	30,100	2.62	100	—	—	^δ^ T65G, T203Y, T205S
**sYFP2**	509	522	38,200	0.09	3,440	0.74	22	2.9	78	^δ^ T65L, T203Y, T205S
**sYFP3**	515	524	37,100	0.35	12,990	0.47	40	3.28	60	^δ^ T203Y, T205A
**sCFP1**	433	475	20,400	0.18	3,670	0.81	40	2.4	60	^δ^ D19E, D21E, Y66W, E124V, H148D, T205S
**sCFP2**	433	476	21,300	0.23	4,900	0.64	25	2.52	75	^δ^ D19E, D21E, Y66W, H148D, V167I, T205S

As a result, in the Results section under the subheading ‘Point mutations to generate full-length and split-GFP, split-YFP and split-CFP variants’,

“We also produced two flCFP/sCFP variants^21^, flCFP1/sCFP1 (D19E/D21E/Y66W/E124V/ H148D/T205S) and flCFP2/sCFP2 (D19E/D21E/Y66W/E124V/H148D/V167I/T205S) (Table 1). Both variants contain the Y66W substitution that alters the GFP spectral properties to CFP^25^, the H148D and T205S substitution that improve CFP quantum yield (QY)^26,27^, a E124V substitution, which allows for faster folding rates in superfolder GFP^28^ as well as D19E and D21E substitutions that provide faster initial rate of cyan fluorescence appearance compared to sCFP carrying only Y66W and T205S mutations^21^. flCFP2/sCFP2 variants also contain a V167I substitution, which provides increased cyan fluorescence brightness^21^.”

should read:

“We also produced two flCFP/sCFP variants^21^, flCFP1/sCFP1 (D19E/D21E/Y66W/E124V/ H148D/T205S) and flCFP2/sCFP2 (D19E/D21E/Y66W/H148D/V167I/T205S) (Table 1). Both variants contain the Y66W substitution that alters the GFP spectral properties to CFP^25^, the H148D and T205S substitutions that improve CFP quantum yield (QY)^26,27^, and the D19E and D21E substitutions that provide faster initial rate of cyan fluorescence appearance compared to sCFP carrying only Y66W and T205S mutations^21^. The flCFP1/sCFP1 variants contain a E124V substitution, which reportedly allows for faster folding rates in superfolder GFP^28^, while the flCFP2/sCFP2 variants contain a V167I substitution, which provides increased cyan fluorescence brightness^21^.”

In the Results section under the subheading ‘Spectral properties of full-length FP and split-FP variants’,

“The additional V167I substitution in flCFP2/sCFP2 did not induce significant spectral changes (Table 1, Fig. 1a,b).”

should read:

“The additional V167I substitution and the absence of E124V in flCFP2/sCFP2 did not induce significant spectral changes (Table 1, Fig. 1a,b).”

In the Results section under the subheading ‘Photobleaching characteristics of split-GFP variants’,

“Concerning the photobleaching rates (*k*_*3*_), the V167I mutation in sGFP1 slows down irreversible photobleaching by 15% compared to sGFPori.”

should read:

“Concerning the photobleaching rates (*k*_*3*_), the V167T mutation in sGFP1 slows down irreversible photobleaching by 15% compared to sGFPori.”

In the Results section under the subheading ‘Folding kinetics of full-length FP variants’,

“Interestingly, the additional V167I mutation in flCFP2 dramatically improves both *k*_*fold1*_ (1.955 min^−1^) and *k*_*fold2*_ (0.195 min^−1^) compared to flCFP1.”

should read:

“Interestingly, the additional V167I mutation and the absence of E124V in flCFP2 dramatically improve both *k*_*fold1*_ (1.955 min^−1^) and *k*_*fold2*_ (0.195 min^−1^) compared to flCFP1.”

In the Results section under the subheading ‘Chromophore maturation kinetic of full-length FP variants’,

“The flCFP variants flCFP1 (*k*_*mat*_: 0.011 min^−1^) and flCFP2 (*k*_*mat*_: 0.012 min^−1^) have maturation rate constants similar to that previously reported for ECFP (*k*_*mat*_: 0.0096 min^−1^)^55^ and the V167I substitution in flCFP2 does not impact the maturation rate (Fig. 3c).”

should read:

“The flCFP variants flCFP1 (*k*_*mat*_: 0.011 min^−1^) and flCFP2 (*k*_*mat*_: 0.012 min^−1^) have maturation rate constants similar to that previously reported for ECFP (*k*_*mat*_: 0.0096 min^−1^)^55^ and neither the presence of E124V in flCFP1 nor that of V167I in flCFP2 impact the maturation rate (Fig. 3c).”

