# Supplementary material for: Characterization of Split Fluorescent Protein Variants and Quantitative Analyses of Their Self-Assembly Process
Source: Sci Rep. 2018 Mar 28;8:5344. doi: 10.1038/s41598-018-23625-7 (PMC5871787; doi:10.1038/s41598-018-23625-7)
Supplement: Supplementary file 1 — Supplementary Information [file 41598_2018_23625_MOESM1_ESM.pdf]

SUPPLEMENTARY INFORMATION

Characterization of Split Fluorescent Protein Variants and Quantitative Analysis of Their Self-Assembly Process

Tugba Koker[1], Anthony Fernandez[1], Fabien Pinaud[1,2,3*]

[1]Department of Biological Sciences, University of Southern California
[2]Department of Chemistry, University of Southern California
[3]Department of Physics and Astronomy, University of Southern California

*Correspondence should be addressed to F.P. (pinaud@usc.edu)

Equilibrium denaturation assays

For equilibrium denaturation, flFPs and complemented sFPs at 1 µM were incubated in duplicate with increasing concentrations of guanidine hydrochloride in TNG buffer pH 8.0 at 25˚C and using a concentration range of guanidine hydrochloride from 0-6 M in 0.4 M intervals. After 96 hours incubation, where equilibrium between folded and unfolded states is reached, changes in FP fluorescence were measured in 96 well-plates on a Biotek Synergy H4 microplate reader with appropriate excitation/emission filters (452DF17/48DF10 nm for CFP, 485DF20/528DF20 nm for GFPs and 500DF13/536DF10 nm for YFP). Data are reported in **Supplementary Fig. S1** as percentage of unfolded FPs as a function of guanidine hydrochloride concentration. To facilitate a visualization of transition regions, denaturation curves are fitted with a two-state folded/unfolded model[1] (F↔U) for flFPs and a three-state folded/intermediate/unfolded model[1] (F↔I↔U) for complemented sFPs where F is the folded state, U is the unfolded state and I is an intermediate state corresponding to the early detachment of the 11th β-sheet M3 peptide in complemented sFPs under denaturing conditions.

Photobleaching kinetic analysis

The photobleaching kinetics in **Figure 2b** were fitted by the solution of the following differential equations[2]:

$$d[FP_{nat}]/dt = -(k_1 + k_3)[FP_{nat}] + k_2[FP_{rble}]$$
$$d[FP_{rble}]/dt = -(k_2)[FP_{rble}] + k_1[FP_{nat}] \qquad (1)$$

where $[FP_{nat}]$ represents the concentration of FPs in their native state and is assumed to be $[FP_{nat}] = 1$ at initial conditions, $[FP_{rble}]$ represents the concentration of reversibly bleached FPs and is assumed to be $[FP_{rble}] = 0$ at initial conditions, k_1 represents the forward rate constant toward the photoconvertible dark state, k_2 represents the backward rate constant from the photoconvertible dark state toward the native FP state, and k_3 represents the rate constant of irreversible photobleaching. We assumed that $[FP_{ible}] = 0$ at initial conditions[2].

Anti-GFP immunostaining of GPI-sCFPs expressed in U2OS

U2OS cells transfected with cDNA encoding GPI-sCFP fusion were briefly washed twice with warm PBS and fixed with 4% PFA in PBS at RT for 15 minutes. Cells were then permeabilized with 0.1% Triton X-100 for 15 minutes and blocked with 4% BSA + 0.1% Tween-20 for 30 minutes. Cells were stained using

a 1:1000 dilution of rabbit anti-GFP Alexa Fluor 647 conjugate (Invitrogen) in blocking buffer for 45 minutes, washed and imaged by confocal fluorescence microscopy (**Supplementary Fig. S3**).

BS3 crosslinking assay and immunoblot analysis.

U2OS cells stably expressing GPI-sGFP2 were used to assess the oligomeric state of sFP fusions in cells. Briefly, cells were rinse with cold PBS and incubated with or without 2 mM bis(sulfosuccinimidyl) suberate (BS3) for 30 minutes on ice, before quenching the cross-linking reaction with 20 mM Tris pH 8.0 for 15 minutes. After BS3 quenching cells were treated for 10 min with or without 20 mM N-ethylmaleimide (NEM), a disulfide bond reducing agent included to verify that possible disulfide bond formations between denatured GPI-sGFP2, which contains two cysteines, do not induce post-extraction multimerization. After cell scraping and lysis in a cell homogenizer at 4°C, cell extracts were run on a denaturing SDS-page electrophoresis gel, before transfer on PVDF immunoblotting membranes (Biorad) and blocking with 5% dry milk in TBST buffer (20 mM Tris-HCl, 150 mM NaCl, pH 7.4 with 0.05 % tween-20). Detection of GPI-sGFP2 was performed with a monoclonal mouse anti-GFP (JL-8) primary antibody (Clontech 632381, 1:2000 dilution) and an HRP-conjugated polyclonal goat anti-mouse secondary antibody (Invitrogen 31430, 1:3000 dilution). Photoluminescent signals were detected on a Biorad Chemidoc using a SuperSignal West Pico chemiluminescent substrate (ThermoFisher).

As shown in **Supplementary Figure S4**, under conditions where no BS3 or NEM are applied, GPI-sGFP2 is detected at about 32 kDa, a molecular weight similar to that expected for the fusion (theoretical molecular weight is 32 kDa). While the detected bands are wide because of gel retardation effects due to the GPI-anchor, there is no other higher molecular bands, indicating that GPI-sGFP2 is primarily monomeric under denaturing electrophoresis condition, as expected. The addition of NEM does not induce changes in band position, indicating that there is little-to-no effect of disulfide bond formation on the oligomeric state of GPI-sGFP2. Under BS3 cross-linking conditions, a monomeric band of GPI-sGFP2 is still observed, but it is accompanied with higher molecular weight bands in the range of 160-200 kDa, much higher than expected for GPI-sGFP2 dimers. Indeed, there was no signal detected at the expected ~64 kDa position for the GPI-sGFP2 dimers indicating that BS3 cross-linking does not result in dimer enrichment and that the GPI-sFP fusions do not form significant dimers in cells. The observed high molecular weight bands of GPI-sGFP2 likely stem from its random cross-linking to other nearby membrane proteins. Combined BS3 and NEM treatments did not induce changes in band positions.

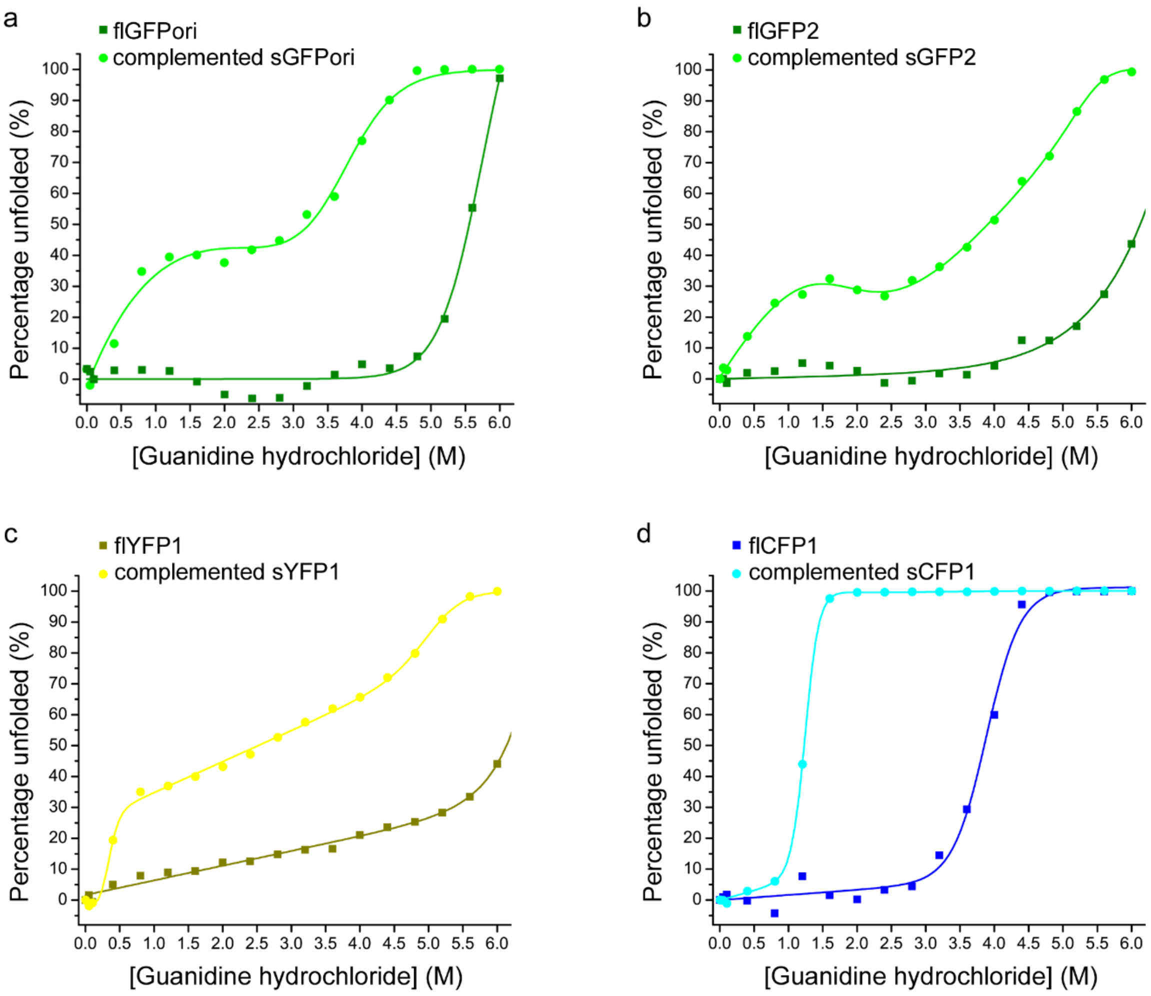

Figure S1: Equilibrium denaturation of flFPs and complemented sFPs at increasing concentrations of guanidine hydrochloride denaturant. a) Comparison of chemical denaturation curves between flGFPori and complemented sGFPori. b) Comparison of chemical denaturation curves between flGFP2 and complemented sGFP2. c) Comparison of chemical denaturation curves between flYFP1 and complemented sYFP1. d) Comparison of chemical denaturation curves between flCFP1 and complemented sCFP1. Denaturation curves are fitted with a two-state folded/unfolded model for flFPs or a three-state folded/intermediate/unfolded model to facilitate a visualization of the transition region. Complemented sFPs systematically display higher sensitivities to guanidine hydrochloride denaturation compared to flFPs. This is consistent with complemented sFPs having a less sturdy β-barrel structure and the possibility that, in the folded state, their chromophore is more exposed to the surrounding environment as suggested by their apparent shorter fluorescence lifetime compared to flFPs.

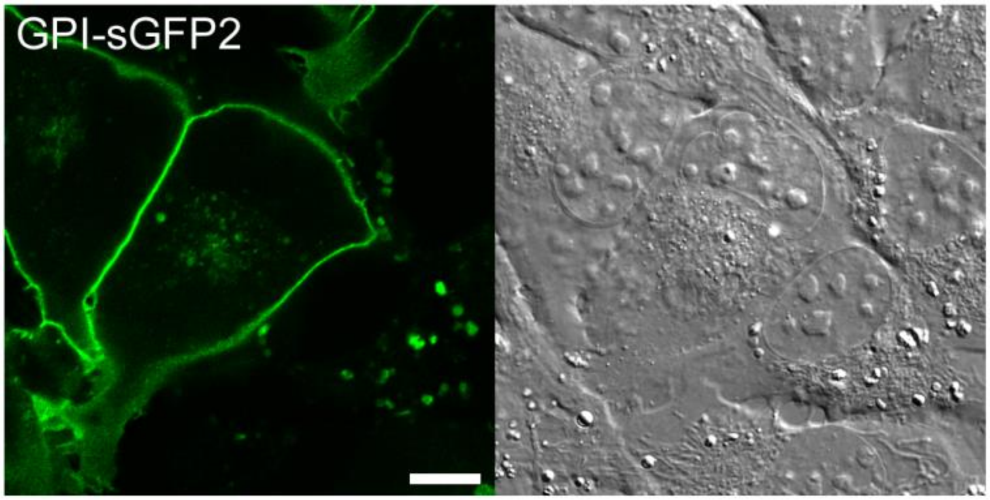

Figure S2. Cross-section confocal and DIC images of U20S cells expressing GPI-sGFP2. Fluorescence complementation is observed only at the cell plasma membrane of expressing cells. Scale bar 10 µm.

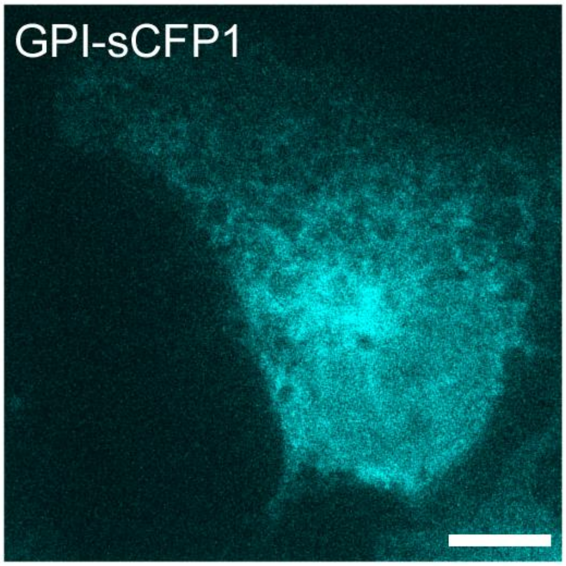

Figure S3. Immunostaining of GPI-sCFP1 expressed in U2OS cells. The staining pattern of GPI-sCFP1 indicates that is it primarily retained in the endoplasmic reticulum, suggesting an ineffective translocation of the fusion protein to the plasma membrane, contrary to other GPI-sGFP and GPI-sYFP fusions. GPI-sCFP2 was similarly retained in the endoplasmic reticulum. Scale bar: 15 μm.

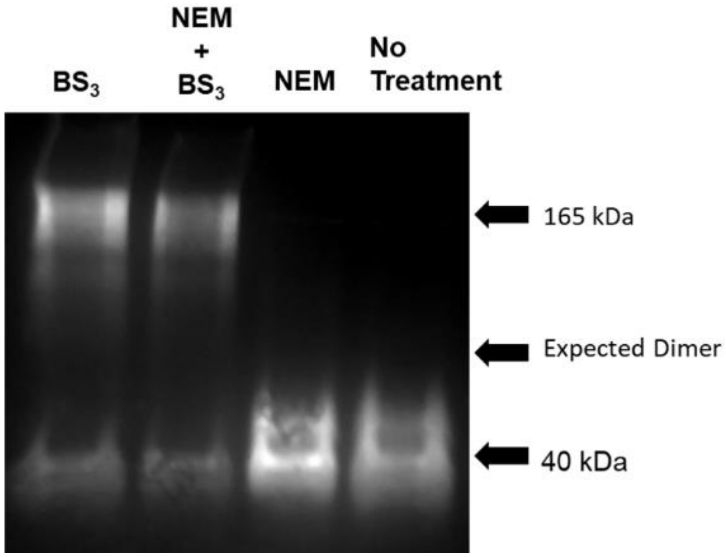

Figure S4. Immunoblot analysis of GPI-sGFP2 oligomerization at the plasma membrane of U2OS cells.

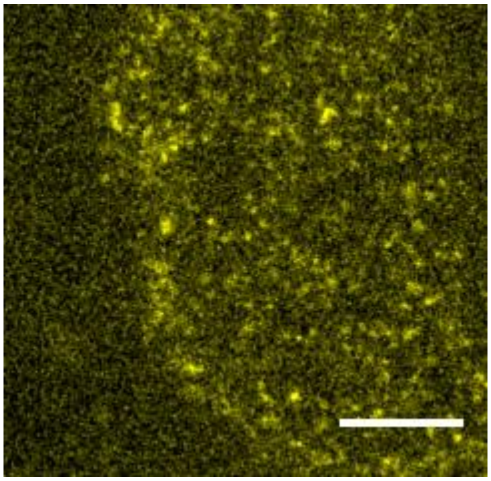

Video V1. Total internal reflection fluorescence imaging of individual complemented GPI-sYFP3 diffusing at the plasma membrane of a U2OS cell. Acquisition and video playback: 25 frame per seconds. Scale bar: 10 µm.

REFERENCES

1 Dignam, J. D., Qu, X. & Chaires, J. B. Equilibrium unfolding of Bombyx mori glycyl-tRNA synthetase. *J. Biol. Chem.* **276**, 4028-4037 (2001).

2 Sinnecker, D., Voigt, P., Hellwig, N. & Schaefer, M. Reversible Photobleaching of Enhanced Green Fluorescent Proteins. *Biochemistry* **44**, 7085-7094 (2005).
